# Aging, mortality, and the fast growth trade-off of *Schizosaccharomyces pombe*

**DOI:** 10.1371/journal.pbio.2001109

**Published:** 2017-06-20

**Authors:** Hidenori Nakaoka, Yuichi Wakamoto

**Affiliations:** 1 Department of Basic Science, Graduate School of Arts and Sciences, University of Tokyo, Komaba, Meguro-ku, Tokyo, Japan; 2 Research Center for Complex Systems Biology, University of Tokyo, Komaba, Meguro-ku, Tokyo, Japan; Newcastle University, United Kingdom of Great Britain and Northern Ireland

## Abstract

Replicative aging has been demonstrated in asymmetrically dividing unicellular organisms, seemingly caused by unequal damage partitioning. Although asymmetric segregation and inheritance of potential aging factors also occur in symmetrically dividing species, it nevertheless remains controversial whether this results in aging. Based on large-scale single-cell lineage data obtained by time-lapse microscopy with a microfluidic device, in this report, we demonstrate the absence of replicative aging in old-pole cell lineages of *Schizosaccharomyces pombe* cultured under constant favorable conditions. By monitoring more than 1,500 cell lineages in 7 different culture conditions, we showed that both cell division and death rates are remarkably constant for at least 50–80 generations. Our measurements revealed that the death rate per cellular generation increases with the division rate, pointing to a physiological trade-off with fast growth under balanced growth conditions. We also observed the formation and inheritance of Hsp104-associated protein aggregates, which are a potential aging factor in old-pole cell lineages, and found that these aggregates exhibited a tendency to preferentially remain at the old poles for several generations. However, the aggregates were eventually segregated from old-pole cells upon cell division and probabilistically allocated to new-pole cells. We found that cell deaths were typically preceded by sudden acceleration of protein aggregation; thus, a relatively large amount of protein aggregates existed at the very ends of the dead cell lineages. Our lineage tracking analyses, however, revealed that the quantity and inheritance of protein aggregates increased neither cellular generation time nor cell death initiation rates. Furthermore, our results demonstrated that unusually large amounts of protein aggregates induced by oxidative stress exposure did not result in aging; old-pole cells resumed normal growth upon stress removal, despite the fact that most of them inherited significant quantities of aggregates. These results collectively indicate that protein aggregates are not a major determinant of triggering cell death in *S*. *pombe* and thus cannot be an appropriate molecular marker or index for replicative aging under both favorable and stressful environmental conditions.

## Author summary

Multicellular organisms universally senesce and must produce rejuvenated progenies in order to transmit life. Although similar age-related deterioration in physiological functions and reproduction is also found in unicellular organisms that divide asymmetrically to produce morphologically distinct aged and younger cells, it has been unclear whether symmetrically dividing microbes—such as fission yeast—exhibit the same traits. Using long-term live-cell microscopy combined with a microfluidic device, we monitor the growth and death of a large number of fission yeast cells and demonstrate the existence of aging-free lineages. These lineages are, however, not immortal, and the probability of death increases as the cells grow more rapidly; thus, the “live fast, die fast” trade-off exists in fission yeast. We further characterize the segregation and inheritance of protein aggregates, which are commonly thought of as “aging factors.” The aging-free lineages bear the aggregate load for some generations with no apparent adverse effects on growth. We also show that there is no threshold amount of protein aggregate above which cells are destined to death in both normal and stressed conditions: protein aggregate is thus not a direct initiation signal for cell death. Our data reveal that protein aggregation might not be an appropriate index for aging and that we should revisit its role in cell physiology.

## Introduction

Replicative aging in unicellular organisms is defined by a gradual increase in generation time and probability of death as cell divisions increase. In cases of asymmetrically dividing unicellular organisms such as *Caulobacter crescentus*, *Saccharomyces cerevisiae*, and *Candida albicans*, aging is manifested and linked to morphological asymmetry [1–3]. The situation, however, is less clear for symmetrically dividing organisms. While some evidence suggests replicative aging in old-pole cell lineages of *Escherichia coli* [4–6], Wang et al. reported that growth rates of *E*. *coli* old-pole cells did not significantly alter over 200 generations, despite the gradual increases in filamentation and death rates [7]. For the symmetrically dividing fission yeast *S*. *pombe*, earlier studies suggested replicative aging by observation of asymmetry in cell volume at divisions followed by the deaths of the larger cells and asymmetric segregation of carbonylated proteins (one of the biomarkers of oxidative stress). Additionally, it was suggested that inheritance of carbonylated proteins and a birth scar might inversely correlate with survival probability [8–10]. In a more recent study, however, Coelho et al. showed that potential aging factors such as an old pole, a new spindle pole body, and protein aggregates did not correlate with generation time, suggesting that *S*. *pombe* does not age, at least under favorable conditions [11].

The key mechanism to generate aging lineages (and their rejuvenated counterparts) is thought to be asymmetric segregation of “aging factors,” regardless of the mode of cell division. Among the potential aging factors are aggregates of misfolded proteins [12,13]. In *E*. *coli*, naturally occurring protein aggregates reside exclusively at old-pole ends, probably because of nucleoid occlusion, and a negative correlation between the aggregate burden and growth rate is observed [5,6]. Likewise, in *S*. *cerevisiae*, protein aggregates are preferentially found in aging mother cells. Active mechanisms that have been suggested to contribute to asymmetric segregation include organelle-associated confinement and actin cable-dependent retrograde flow [14–17]. Asymmetric damage segregation and its association with aging were also suggested in *S*. *pombe* cultured under heat or oxidative stress conditions [11]. Although it was previously shown how aggregates are formed, move, and segregate during cell division, it is not clearly established whether they contribute to an increased death rate [11,18].

To study the aging process of yeasts, lineage tracking on an agar plate is conventionally performed [19]. This requires micromanipulation to remove daughter cells and is relatively labor intensive, thus precluding high-throughput and long-term analyses. An increasing number of studies at the single-cell level for various model organisms utilize microfluidic devices made of polydimethylsiloxane (PDMS), a chemically stable and biocompatible silicone, in combination with automated microscopic imaging techniques [20–25]. One such device, termed the “Mother Machine,” was originally developed to track *E*. *coli* old-pole cell lineages with considerably higher throughput (10^5^ individual old-pole cells) and longer duration (200 generations) than previous studies [7]. Mother Machine-like microfluidic devices for *S*. *pombe* have been reported recently, and they demonstrated the absence of replicative aging in rich medium [26,27].

In this work, we measured more than 1,500 fission yeast old-pole cell lineages up to 80 generations using a custom-built Mother Machine-like microfluidic device. By measuring cell division and death rates in 7 different balanced growth conditions, we confirmed the absence of replicative aging in old-pole cell lineages in all of the tested environments and found a positive correlation between the division and death rates. We observed formation, growth, inheritance, and asymmetric segregation of Hsp104-associated protein aggregate in the old-pole lineages and demonstrated that inheritance and quantity of protein aggregate affected neither generation time nor triggering of cell death. In addition, a large amount of protein aggregate induced by transient stress treatment could also be tolerated without affecting cellular growth rates. Collectively, our results suggest that protein aggregate does not serve as an aging marker under both favorable and stressful conditions.

## Results

### Old-pole cell lineages exhibit aging-free growth over tens of generations in a microfluidic device

**Fig 1:**
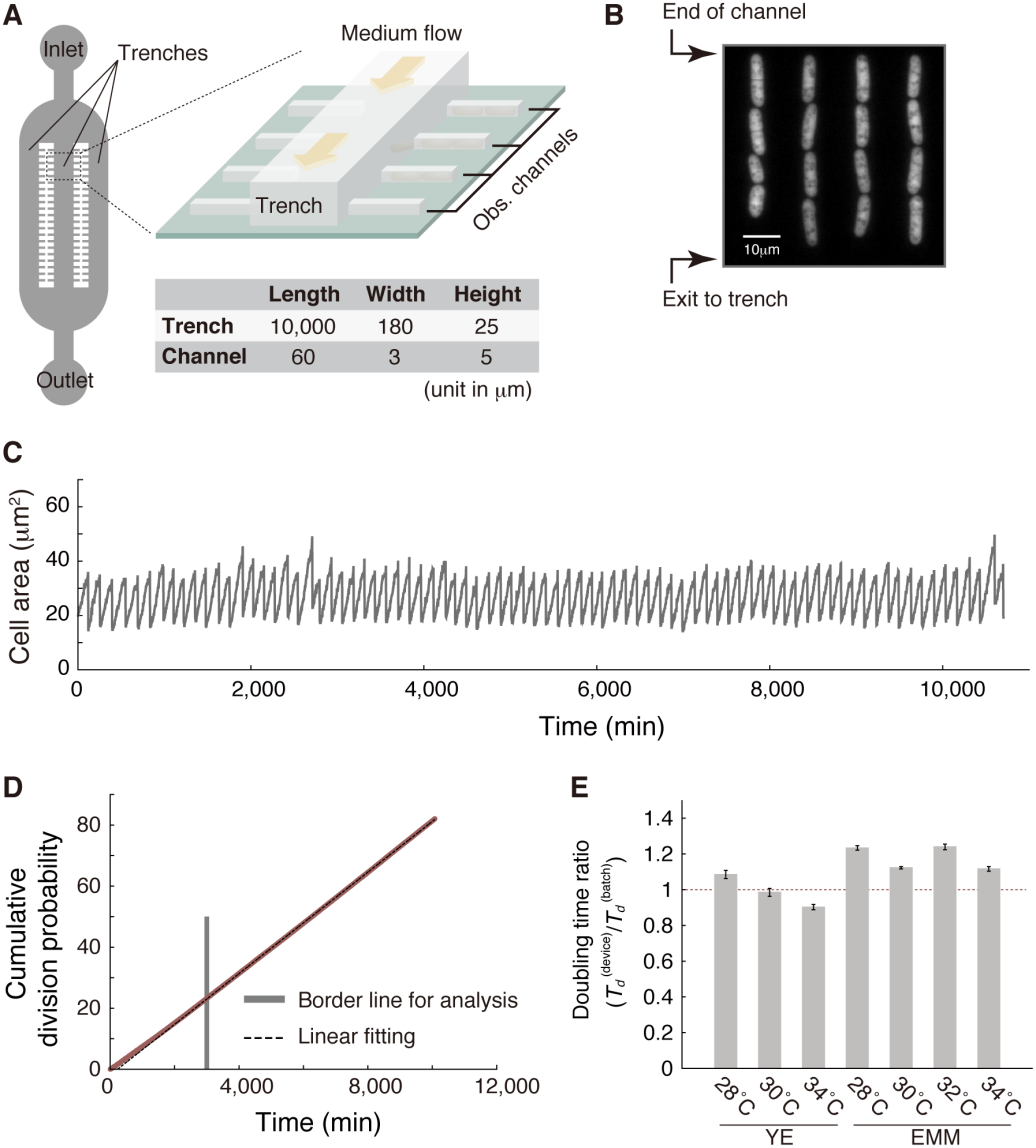
Stability of cell division rate in the microfluidic device. (A) Schematic representation of the device (not to scale, for clarity). Approximate dimensions of the trenches and observation channels are presented in the table. (B) An example of a fluorescence image of yeast cells expressing mVenus loaded into the observation channels. (C) Trajectory of cell size of a single representative lineage in yeast extract medium (YE) at 30°C. (D) The cumulative division probability in YE at 34°C, plotted against time (red). Linear fitting (black broken line) was performed using the time window (*t* ≥ 3,000 min in this example, indicated by a gray vertical line) in which stable growth was achieved. See also S3 Fig. (E) Estimated population doubling times in the microfluidic device (*T*_*d*_^(device)^) relative to those in batch cultures (*T*_*d*_^(batch)^). EMM, Edinburgh minimal medium. See Materials and methods for estimation of the population doubling times from the generation time distributions. The numerical values for the plots are deposited in the Dryad repository: http://dx.doi.org/10.5061/dryad.s2t5t/15.

We designed and developed a microfluidic device for long-term tracking of old-pole cell lineages of *S*. *pombe* (Fig 1A and 1B and S1 Fig). Our device has essentially the same architecture as the “Mother Machine,” which was originally developed by Wang et al. for studying aging and growth in *E*. *coli* [7], except that the dimensions of the internal channels were scaled up for fission yeast, which are physically larger. During time-lapse experiments, the device was constantly supplied with fresh medium to keep the environmental conditions around the cells unchanged. We experimentally confirmed that the medium reached the ends of the observation channels within 5 min, both in the absence and presence of cells (S2 Fig and S1 Movie). Cells grew and divided aligned in the observation channels, and cells that spilled out from the observation channels to the trench were washed out by the flow of medium (Fig 1B and S2 Movie). These settings allowed us to follow the division dynamics of cells located at the ends of the observation channels, referred to as old-pole cells (or mother cells), typically for 50–80 generations. Time-series data on cell size (determined by visualized cell area) for every cell lineage were extracted from a set of time-lapse images (Fig 1C). We analyzed more than 1,500 single-cell lineages in each experiment, which is comparable to, or larger than, the numbers of cell lineages analyzed in similar microfluidic experiments [7,27].

We performed time-lapse experiments employing 7 different culture conditions with different media (yeast extract medium [YE] or Edinburgh minimal medium [EMM]) and temperatures (see S1 Table for the summary of all measurements). Plotting the cumulative division probability against time confirmed that division rates were strikingly stable except during the initial measuring (Fig 1D and S3 Fig). This early instability in division rates reflected a lag in cell recovery from the slow-growing state that follows the loading of the cells into the microfluidic device (see Materials and methods). Population doubling times calculated from the distributions of generation times (see [28–30] for reference) were close to those determined in batch culture experiments in the same media and temperature conditions (Fig 1E and S2 Table). This indicates that the medium exchange rate in the device is sufficiently high. The stability in division rates for 50–80 generations, in turn, suggests an absence of deterioration in the reproductive ability of old-pole cells, under favorable culture conditions.

**Fig 2:**
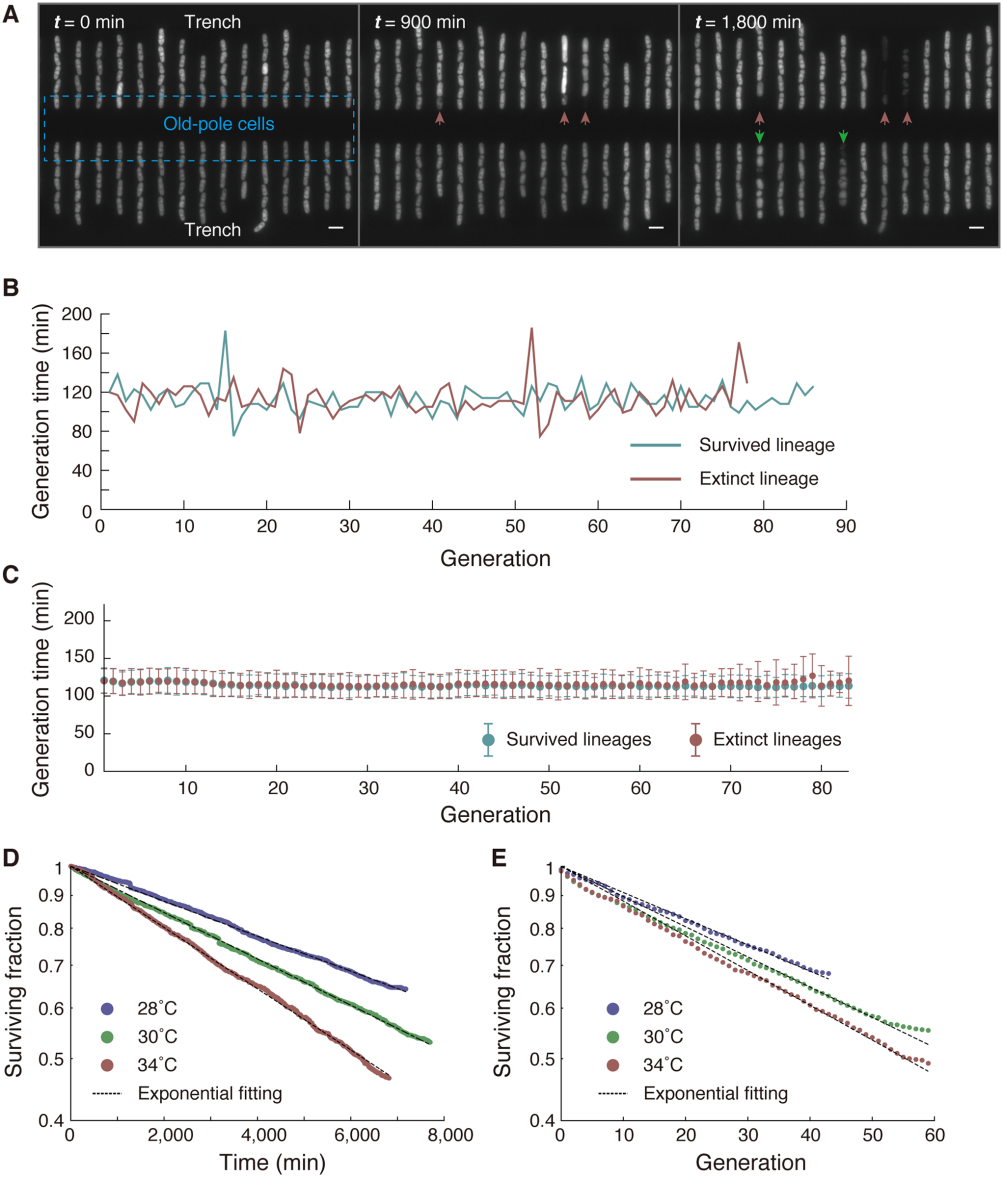
Characterization of cell deaths in constant environments. (A) Three fluorescence images of the same position at different times in yeast extract medium (YE) at 34°C. While all of the old-pole cells were alive at the beginning (*t* = 0), 3 died by 900 min (red arrows), and 2 others by 1,800 min (green arrows). Scale bars indicate 10 μm. (B) Representative generation time transitions in surviving (blue) and extinct (red) lineages in YE at 34°C. (C) Transition of mean generation time for each generation in YE at 34°C. Error bars represent standard deviations. See also S5 Fig. (D) Decay of the surviving fraction of old-pole cell lineages against time in YE at 3 temperature conditions (blue, 28°C; green, 30°C; and red, 34°C). See also S6 Fig. (E) Decay of surviving fractions plotted against generation count. The numerical values for the plots are deposited in the Dryad repository: http://dx.doi.org/10.5061/dryad.s2t5t/15.

Despite these favorable growth conditions, we observed the deaths of individual cells at low frequencies over the entire observation period (Fig 2A, S2 Movie and S1 Table). Because the death events were observed throughout the time-lapse experiments and in every imaged position, they were not caused by temporal and/or local alterations in culture environments. The behaviors of cells destined for death were heterogeneous but could be broadly categorized into 3 types: Type I (swollen), Type II (hyperelongated), or Type III (shrunken). Approximately 80% of the death events were categorized as Type I, and in almost all of these cases, siblings in the same observation channel synchronously died (S4D and S4E Fig and S2 Movie). These observations are consistent with a recent report using a similar microfluidics system [27]. The synchronous deaths were also observed in another PDMS microfluidic device, where the observation channels accommodate greater numbers of cells than the Mother Machine. Importantly, we observed the synchronous deaths even when the dying siblings were spatially separated, whereas the other surrounding cells continued dividing normally (S4F Fig and S3 Movie). These findings suggest that the synchronous deaths are not induced by local environmental changes in channels but are triggered in their common ancestor cells.

We did not detect any preceding progressive signatures in the growth and division histories of dead cells. For example, the transitions in generation times of the extinct cell lineages were indistinguishable from those of the surviving lineages; no obvious or discernible increase in generation times was observed prior to cell deaths (Fig 2B). The stability of generation times in the extinct cell lineages was further confirmed by comparing the means and standard deviations of generation times for each generation between the surviving and extinct lineages (Fig 2C and S5 Fig). In addition, the number of surviving cell lineages decayed exponentially with time and generation count (Fig 2D and 2E and S6 Fig), indicating that cell deaths occurred randomly with fixed probabilities and that every lineage exhibited an equal chance of abrupt death. The death rates estimated from the decay curves were small, in the order of 10^−5^ per min (or 10^−2^ per generation). We noticed that our standard fluorescence imaging conditions induced weak photodamage [31]. Consequently, the estimated death rates were slightly higher than the death rates obtained by bright field imaging alone, but the constancy of the death rates was unaltered (S4B and S4C Fig).

### Trade-off between reproduction and survival in balanced growth conditions

**Fig 3:**
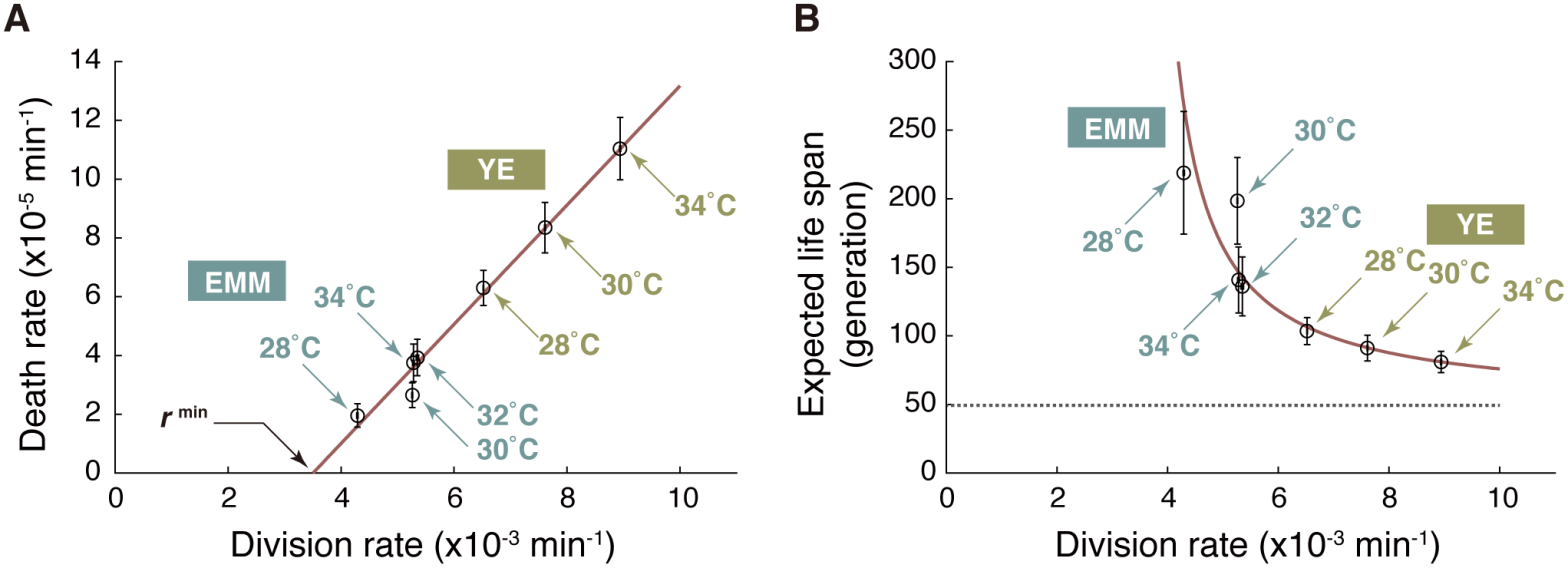
Trade-off between division and death rates. (A) Relationship between division rates (*r*) and death rates (*k*). The red line represents the best linear fit to the data points (open circles) by the least squares method. The error bars represent ±2 standard error ranges (see Materials and methods for the rigorous definitions). (B) Relationship between division rate (*r*) and expected life span (τ_d_/τ_b_ = *r*/*k*) of a single-cell lineage. The red line is a theoretical curve based on linear fitting in (A): *k* = α (*r–r*^min^), where the slope α = (2.0 ± 0.1) × 10^−2^, and *r*^min^ = (3.5 ± 0.2) × 10^−3^ min^-1^. The gray broken line represents the minimum expected life span (1/α) in the fast-growth limit. EMM, Edinburgh minimal medium; YE, yeast extract medium. The numerical values for the plots are deposited in the Dryad repository: http://dx.doi.org/10.5061/dryad.s2t5t/15.

We next investigated how cellular division and death rates might be interrelated. As presented in Fig 3A, we found that the death rate increased linearly with the division rate. The values of each data point (including error estimates) and the corresponding culture conditions are summarized in S3 Table. For example, in YE at 34°C, the division rate was *r* = (8.94 ± 0.01) × 10^−3^ min^-1^ (mean generation time τ_b_ = 1/*r* = 112 min), and the death rate was *k* = (1.1 ± 0.1) × 10^−4^ min^-1^ (characteristic lifetime τ_d_ = 1/*k* = 9.1 × 10^3^ min = 6.3 days). Additionally, in EMM at 28°C, the division rate was *r* = (4.29 ± 0.01) × 10^−3^ min^-1^ (τ_b_ = 233 min), and the death rate was *k* = (2.0 ± 0.4) × 10^−5^ min^-1^ (τ_d_ = 5.0 × 10^4^ min = 35 days). Thus, fast growth significantly shortens the lifetime of single cells. Reformatting the plot reveals that the expected life span of single-cell lineages in units of generation (τ_d_/τ_b_) also decreases with division rate, asymptotically approaching the minimum bound (the shortest expected life span) of approximately 50 generations (Fig 3B, gray broken line). The decrease in expected life span can be attributed to the fact that the death rate reaches 0 with a positive division rate value (*r*^min^ = [3.6 ± 0.2] × 10^−3^ min^-1^, equivalently τ_b_^max^ = 280 min), as shown in Fig 3A.

It should be noted here that Spivey et al. has recently reported that the death probability per generation of *h*^-^ *972* (the same strain used in our study) grown in rich medium was about 1.8% where the mean generation time was about 130 min [27]. These values correspond to the division rate *r* = 7.7 × 10^−3^ min^-1^ and the death rate *k* = 1.4 × 10^−4^ min^-1^. The linear relation in Fig 3A predicts that *k* = 8.6 × 10^−5^ min^-1^ when *r* = 7.7 × 10^−3^ min^-1^, which is 1.6-fold lower than their experimental value but reasonably close despite the different configurations of the microfluidic devices.

### Aggregated protein deposits remain in old-pole cell lineages for multiple generations but are eventually segregated to new-pole cells

**Fig 4:**
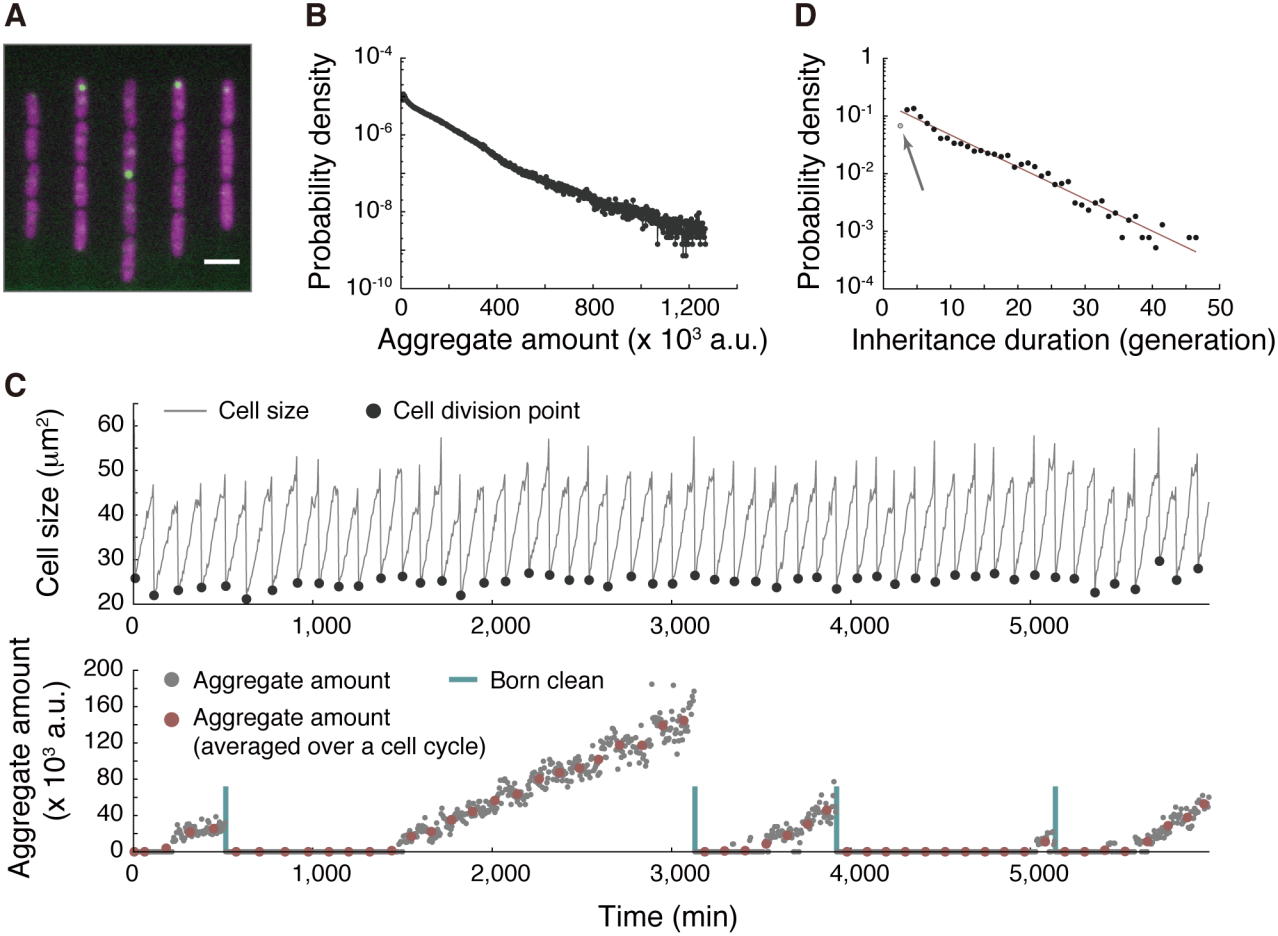
Formation and segregation dynamics of Hsp104-associated protein aggregate. (A) An example of merged fluorescence images (green fluorescent protein [GFP] and red fluorescent protein [RFP] channels) of the strain HN0045 cultured in the microfluidic device. Green: Hsp104-GFP. Magenta: mCherry. Scale bar indicates 10 μm. (B) Distribution of aggregate amounts; *N* = 932,801. (C) Typical cell size trajectory of HN0045 grown in the microfluidic device (top) and dynamics of formation, growth, and segregation of Hsp104-GFP foci in the lineage (bottom); gray closed circles: aggregate amount at each time point; red closed circles: cell-cycle-averaged aggregate amount; blue vertical lines: points of cell divisions that produced aggregate-free old-pole cells. A time interval between 2 adjacent blue lines is defined as duration of aggregate inheritance. (D) Distribution of aggregate inheritance interval. Data points, excluding the gray one indicated by an arrow, were fitted by shifted-exponential distribution: *p*(*x*) = *λe*^−*λ(x*−*μ*)^. A line of best fit (1/λ = 7.8 and μ = 2.1) is shown colored in red. The numerical values for the plots are deposited in the Dryad repository: http://dx.doi.org/10.5061/dryad.s2t5t/15.

Our observation that fission yeast old-pole cell lineages are unlikely to undergo replicative senescence motivated us to proceed to monitor long-term dynamics of protein aggregation to gain insight into how these lineages avoid aging. In general, protein aggregates are associated with molecular chaperones and heat shock proteins that can extricate protein monomers from the aggregates and refold them into their native structures [12,13]. One of the most well-studied heat shock proteins in yeasts is Hsp104, an ATP-dependent disaggregase that is often used as a molecular marker of protein aggregation in both *S*. *cerevisiae* and *S*. *pombe*. A strain that expresses Hsp104–green fluorescent protein (GFP) from the native chromosomal locus was observed in the microfluidic device for approximately 50 generations (Fig 4A and S4 Movie). The time-lapse imaging revealed that most healthy growing cells had 0 or 1 major GFP focus. We defined aggregates as a set of connected pixels whose fluorescence (i.e., GFP) intensity exceeded a defined threshold value and quantified aggregate amounts by integrating fluorescent intensity within the connected area (S7 Fig). The distribution of aggregate amounts was roughly exponential (Fig 4B), which is consistent with previously reported results [18]. Fig 4C presents a representative dynamic of formation, growth, and segregation of protein aggregates in an old-pole cell. Once formed at an old-pole end, the (major) aggregate grew and tended to remain at the pole for many generations, but it occasionally migrated toward the new-pole end and was subsequently segregated to the new-pole cell (S4 Movie), which is qualitatively consistent with an earlier report [18]. The distribution of aggregate inheritance duration, which is defined as the time interval between 2 successive “born-clean events” in units of generation, had a peak at 4 generations with an extended tail to the right and spreading over more than 40 generations (Fig 4D). The tail can be approximately fitted by an exponential curve with a decay rate of λ = 0.13 (generation^-1^), suggesting that the segregation of protein aggregate to a new-pole cell is a random process that occurs once in every 1/λ = 7.8 generations on average. These results revealed that fission yeast old-pole cell lineages could escape from the burden of protein aggregate.

### Protein aggregation affects neither generation time nor initiation of cell death

**Fig 5:**
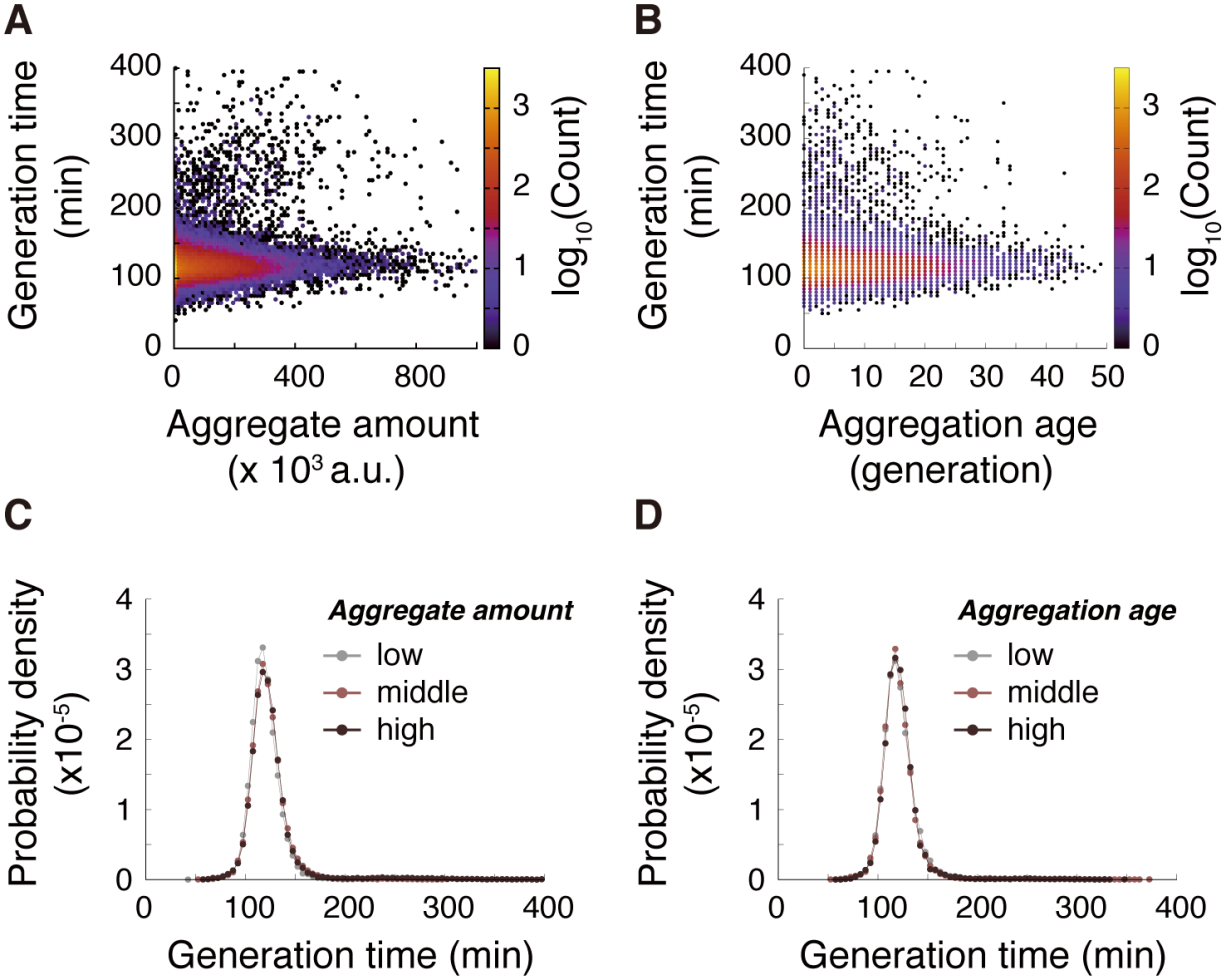
Hsp104-associated protein aggregates do not affect generation time. (A) Correlation between aggregation level and generation time; *N* = 53,683 and Spearman’s ρ = 0.081. (B) Correlation between aggregation age and generation time. The data count associated with each point is indicated by colors; *N* = 41,076 and Spearman’s rank-order correlation coefficient ρ = 0.016. (C) Generation time distributions for different levels of aggregate amount. *N* = 15,551 (aggregate amount ≤ 5,000), 20,438 (5,000 < aggregate amount ≤ 95,000), and 17,806 (aggregate amount > 95,000). (D) Generation time distributions for different levels of aggregation age. *N* = 13,481 (aggregate age ≤ 2), 14,481 (2 < aggregate age ≤ 8), and 13,115 (aggregate age > 8). The numerical values for the plots are deposited in the Dryad repository: http://dx.doi.org/10.5061/dryad.s2t5t/15.

Although the mean generation time of the old-pole cell lineages was stable (Fig 2B and 2C), heterogeneity in each cell cycle length might be related to protein aggregation. We quantified the load of protein aggregation using 2 metrics: (1) aggregate amount and (2) aggregate age, the latter being defined as elapsed time (in units of generation) since the last birth without aggregate inheritance (indicated by “Born clean” bars in Fig 4C). The former evaluates the current load of aggregation, whereas the latter evaluates the burden of possessing the aggregate for prolonged periods. We first simply plotted generation time against (cell cycle-averaged) aggregate amount (Fig 5A) and aggregation age (Fig 5B), detecting no correlations.

To analyze such relations in greater detail, we partitioned the data points in Fig 5A and 5B into 3 classes (low, middle, and high) according to the aggregation metrics (aggregate amount or aggregation age) and compared the generation time distributions among the classes (Fig 5C and 5D). The distributions were essentially identical among the classes for both aggregation indices, which strongly indicates that cell cycle length is unaffected by protein aggregation.

**Fig 6:**
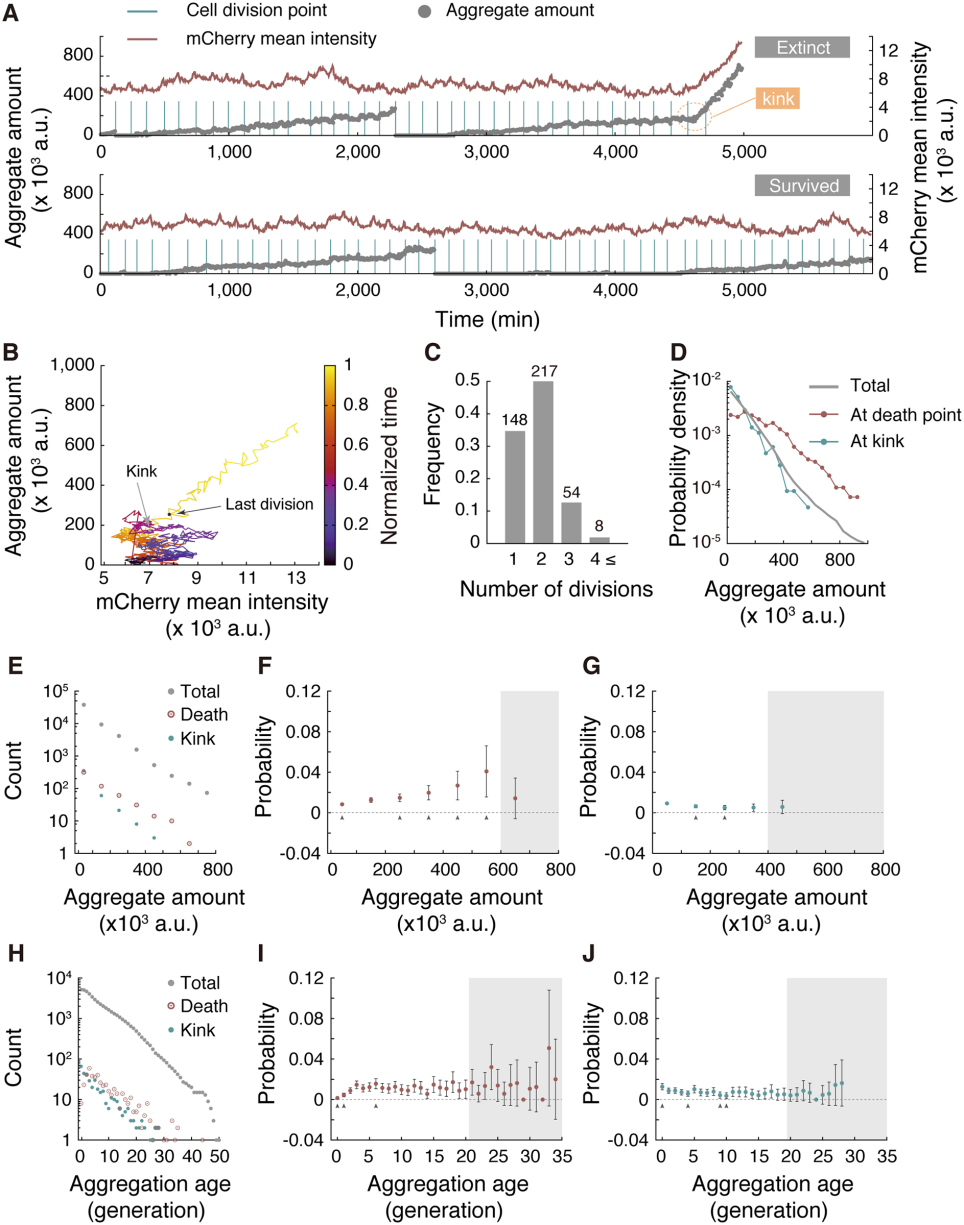
Relationship between Hsp104-associated protein aggregate and death. (A) Typical dynamics of Hsp104-associated protein aggregate (gray closed circles) and constitutively expressed mCherry level (red line) of an extinct lineage (top) and a survived lineage (bottom). The dying process begins around 4,500 min (indicated by a dotted orange circle) in this example, after which both aggregate amount and mCherry signals increase. (B) Dynamics of protein aggregation and mCherry levels plotted in a 2D plane. The data are from the same (extinct) cell lineage shown in (A). Time evolution is indicated by colors from dark blue to yellow. The black and gray points indicate the states at the last division and the kink position, respectively. (C) The number of cell divisions after the kinks until cell death. The figures on top of the bars represent the number of lineages identified. (D) Distributions of aggregate amount at death points (red), kinks (blue), and for all regions of interest (ROIs; gray). (E) Distributions of aggregate amount at birth for all identified cell cycles (gray), for the last generations in extinct lineages (red), and for generations in which the accelerated accumulation (initiation of death) started (blue). (F) Probability of death of cells born with different amounts of aggregate. Gray arrowheads indicate data points whose deviations from the population death probability (*p* = 1.15 × 10^−2^) are statistically significant (binomial test at the significance level α = 0.05. See Materials and methods for details). Data points within the gray-shaded area do not satisfy the commonly employed rule of *Np* ≥ 5 for appropriate normal approximation (*N*, the number of samples) and are excluded from the tests. Error bars show standard errors. The same rule applies to the arrowheads, the gray-shaded area, and the error bars in panels (G), (I), and (J) below. (G) Probability of initiating cell death (= observing a kink) for cells born with different amounts of aggregate. (H) Distributions of aggregation age for all of the ROIs (gray), at death points (red), and at kinks (blue). (I) Death probability at each aggregation age. (J) Probability of observing a kink at each aggregation age. The numerical values for the plots are deposited in the Dryad repository: http://dx.doi.org/10.5061/dryad.s2t5t/15.

Next, we examined if the amount and inheritance of protein aggregation trigger cell death. Fig 6A illustrates protein aggregation dynamics (Hsp104-GFP aggregate amount) along with the level of constitutively expressed protein (mCherry mean fluorescence intensity corresponding to its cellular concentration) for both survived and extinct lineages. Typically, cells destined for death exhibited accelerated accumulation of protein aggregates immediately prior to death (Fig 6A [top panel], after 4,500 min); we detected accelerated accumulation in 79% (427 out of 541) of extinct lineages. The commencement of accelerated accumulation was detectable by clear kinks in the transitions of aggregate amounts. These kinks seem to identify the time of initiation of cell death because other apparent functional deteriorations, such as radical increases in mCherry expression and changes of cellular morphology, also initiated concurrently (Fig 6A and 6B and S4 Movie). Interestingly, even after the onset of these abnormalities, cell division occurred a few times before death (Fig 6C), which might underlie the observed synchronized deaths (S4D, S4F and S4G Fig).

Due to the occurrence of accelerated accumulation in many extinct lineages before deaths, the distribution of aggregates at the death points was shifted toward greater values (Fig 6D). However, the distribution of the amount at the kink points was very close to that for the total population (Fig 6D), which suggests that large aggregate amounts are not required for initiating the process of dying. To reinforce this observation, we counted the numbers of cells that exhibited deaths and kinks for given ranges of aggregate amount (Fig 6E) and evaluated the probability of death (Fig 6F) and of starting accelerated accumulation (Fig 6G). The results showed that the probability of commencing accelerated accumulation did not increase with the aggregate amount, although that of observation of cell deaths was elevated, which again suggests that the aggregate amount is not causative of initiation of the dying processes.

We also investigated whether retention of protein aggregates increased the probabilities of death and of starting accelerated accumulation by counting the numbers of cells that showed deaths and kinks for given aggregation age (Fig 6H). We found that both probabilities were nearly constant, irrespective of aggregation age (Fig 6I and 6J). The death probabilities for aggregation age < 3 generations were slightly lower than the total death probability (1.15 × 10^−2^ per generation) (Fig 6I), possibly because of an identified lag of a few generations before death, after the onset of accelerated accumulation (Fig 6C). Indeed, the probabilities of commencing accelerated accumulation were equally high for these small aggregation-age generations (Fig 6J). Overall, our data suggest that Hsp104-associated protein aggregation is unlikely to play a major role in initiating the dying process. Our results, however, do not exclude the possibility that rapid accumulation of protein aggregate might accelerate completion of the dying processes post onset.

### Protein aggregation induced by oxidative stress does not result in replicative aging

**Fig 7:**
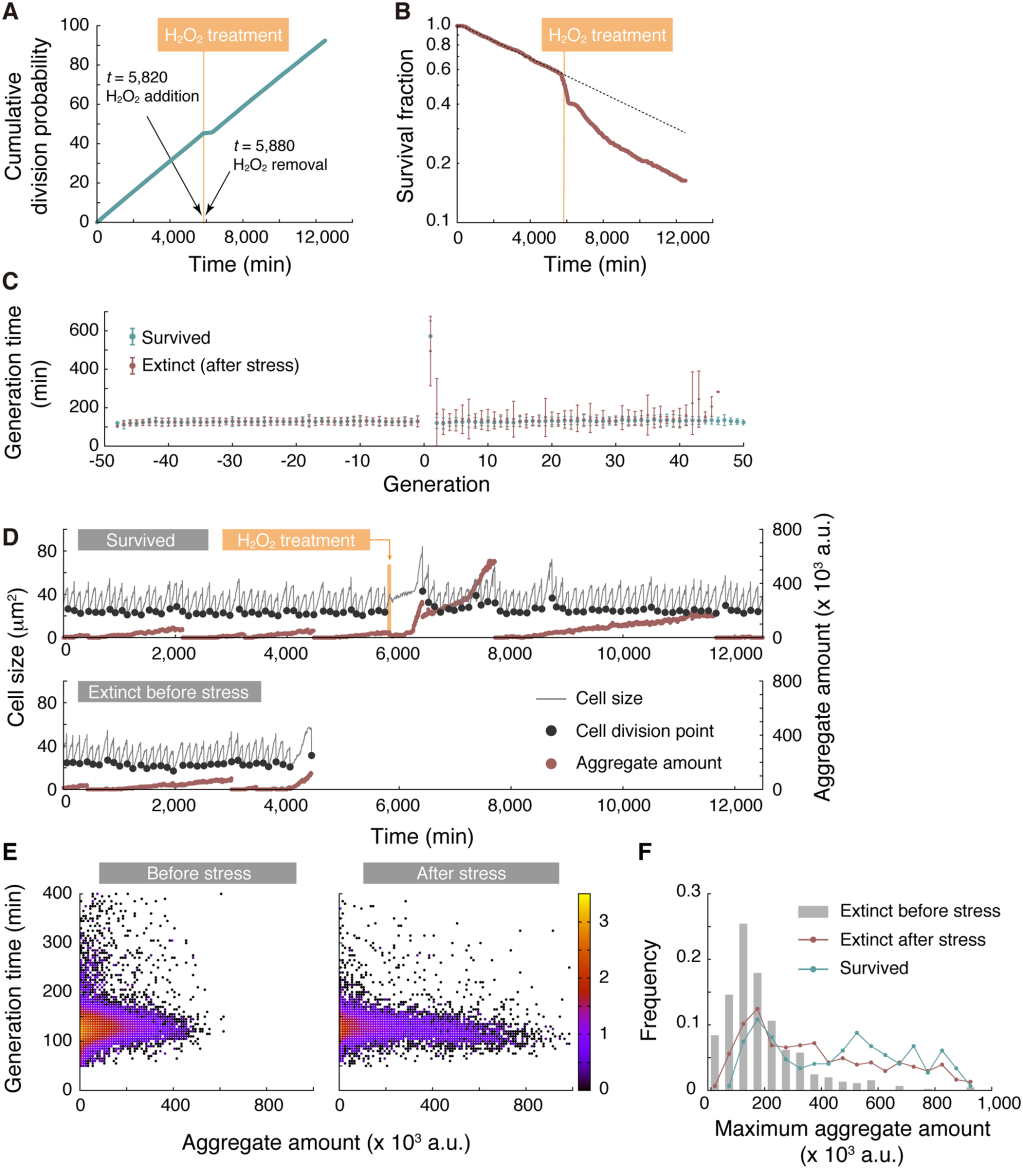
Oxidative stress does not induce replicative aging. (A) Cumulative division probability. Mean generation time, estimated as a reciprocal of the slope of the line, was 130 min before stress and 134 min after recovery. The orange thick line represents the period of H_2_O_2_ treatment (60 min). (B) Survival curve before and after stress treatment. The broken line represents a predicted survival curve without stress treatment. (C) Transitions of mean generation time before and after stress. Positive and negative generations indicate the generations after and before H_2_O_2_ treatment, respectively. Lineages that expired before stress treatment were excluded from the analysis. Error bars show standard deviations. (D) A representative example of a lineage that survived until the end of measurement (top) and a lineage that died before stress treatment (bottom). Stress treatment induces much higher levels of aggregation than in normal conditions. (E) Density maps for the relationship between generation time and aggregate amount. (Left) Before stress treatment. (Right) After stress treatment. Density of points is represented in color (log_10_[Counts]). (F) Distributions of maximum aggregate amount on a lineage. Gray color indicates the lineages that were extinct before stress; red is used for the lineages that were extinct after stress; and blue was used for the survived lineages. The numerical values for the plots are deposited in the Dryad repository: http://dx.doi.org/10.5061/dryad.s2t5t/15.

It has been suggested that fission yeast ages upon stress treatment, and inheritance of large protein aggregate results in increased death probability [11]. To see if these aging phenotypes are also observed in our system, we transiently treated cells with hydrogen peroxide (H_2_O_2_), a commonly used oxidative stressor, and monitored cell division/death kinetics along with protein aggregation dynamics. As expected, cells immediately ceased to divide upon stress treatment (Fig 7A, around *t* = 6,000 min). After removal of the stress, there was a lag (around *t* = 6,000–6,500 min) before cells resumed dividing. Strikingly, once cells started to grow again, the division rate was almost the same as that previously observed under unstressed conditions (Fig 7A). The survival curve in Fig 7B revealed an increase in death rate upon stress treatment (approximately 10% cells died during 1 h of exposure to hydrogen peroxide). Although the recovery was slower than that of division rate, the death rate also returned to the normal level seen in the unstressed condition. We did not observe the progressive increase of generation time after stress removal, one of the hallmarks of replicative aging; marked increase of generation time was seen only in the first generation after stress removal (Fig 7C). These results suggest that the apparent deterioration in cellular growth/death is a transient response to the stress and not a manifestation of aging.

We next asked how protein aggregation dynamics are related to the stress response. As reported earlier, we observed that oxidative stress enhanced protein aggregation, and many lineages accumulated aggregate to high levels not attainable in normal conditions (Fig 7D). When the cells re-entered division cycles after stress removal, the large amount of aggregate persisted (and even continued to grow in some cases). Strikingly, even such significant amounts of aggregate did not affect generation time (Fig 7E). In nonstressed conditions, the amount of aggregate did not exceed 400 (× 10^3^ a.u.) in 90% of extinct lineages, whereas approximately 40% of the lineages that survived the stress treatment until the end of measurement experienced more aggregation than 400 (× 10^3^ a.u.) (Fig 7F). These results further support that the absolute amount of protein aggregate does not determine growth kinetics or cell fates. This contrasts with the previous report [11], in which the authors concluded that *S*. *pombe* exhibits aging under stressful conditions.

### Ectopically induced protein aggregation does not affect cellular growth or death

**Fig 8:**
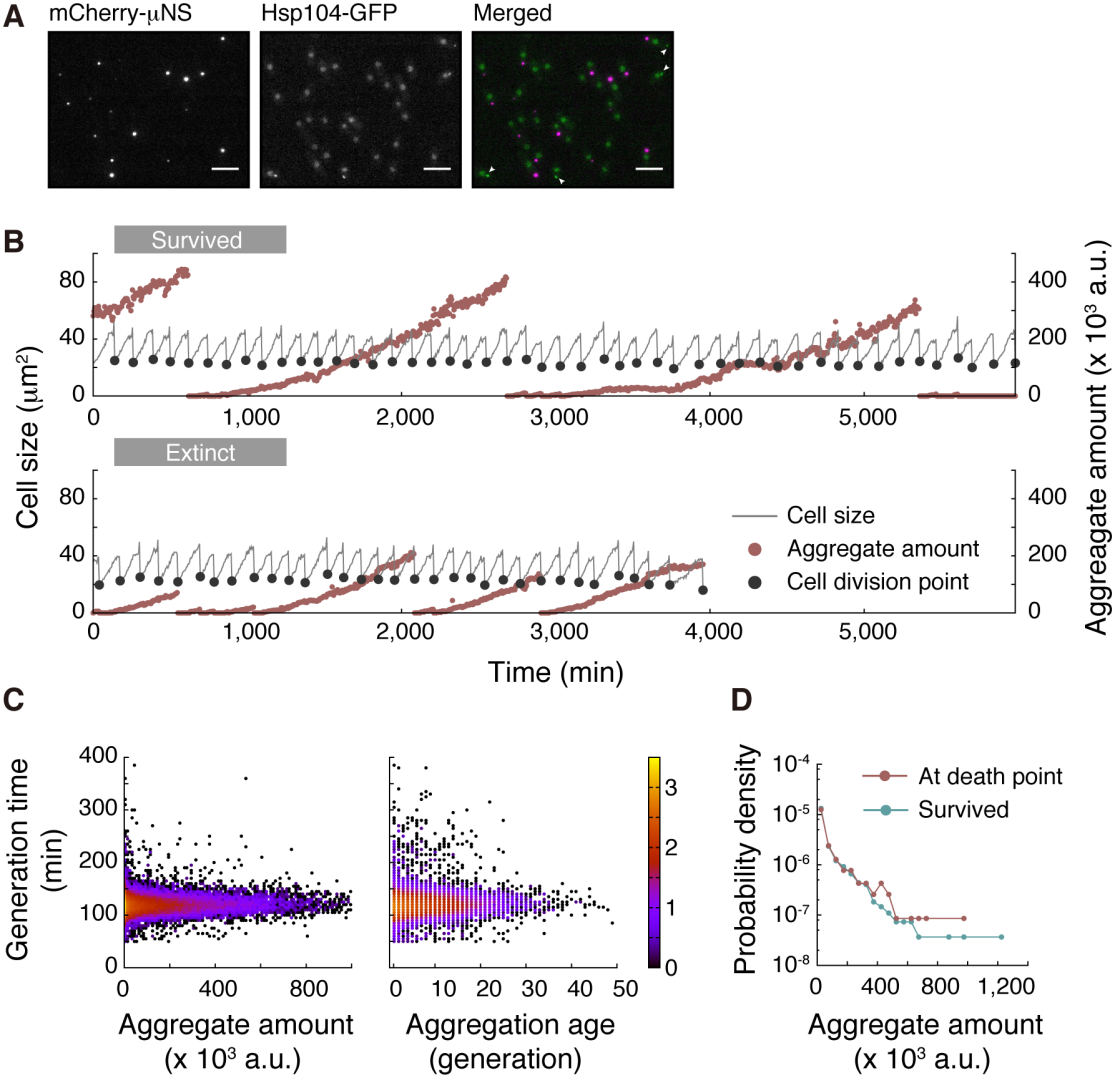
Ectopic expression of aggregation-prone protein does not affect cellular growth and death. (A) μNS and Hsp104 form distinct types of aggregate. mCherry-μNS under the control of the *nmt1P41* promoter was coexpressed with Hsp104–green fluorescent protein (GFP) in Edinburgh minimal medium (EMM) lacking thiamine. (Left) Cytoplasmic foci of mCherry-μNS aggregates. (Middle) Hsp104-GFP aggregates are observed as cytoplasmic foci. In most cases, nuclei are also diffusely stained. (Right) Merged image. No colocalization of μNS and Hsp104 is observed. White arrowheads indicate cytoplasmic Hsp104-GFP foci. Scale bars indicate 10 μm. (B) Representative dynamics of μNS aggregate amounts shown with cell division cycles. (C) No correlation between generation time and aggregate amount (left) and aggregation age (right). (D) Distributions of aggregate amounts at death points (red) and the end point of the survived lineages (blue). The numerical values for the plots are deposited in the Dryad repository: http://dx.doi.org/10.5061/dryad.s2t5t/15.

To further examine if protein aggregation can result in cellular aging and/or cell death in *S*. *pombe*, we ectopically expressed a truncated version of the orthoreovirus aggregation-prone protein, μNS, which was N-terminally tagged with mCherry or mNeonGreen for visualization by fluorescent microscopy [32–34]. We confirmed that μNS formed aggregates in the cytoplasm of *S*. *pombe*, which are detectable as bright foci in many cells (Fig 8A). mCherry-μNS and Hsp104-GFP foci did not colocalize, which indicates that not all protein aggregates were associated with Hsp104 (Fig 8A and S5 Movie). Formation and segregation dynamics of mNeonGreen-μNS aggregate were similar to those of Hsp104-GFP, other than that accelerated accumulation before cell death was not observed (Fig 8B, S8A Fig and S5 Movie). Generation time was not correlated with either aggregate amount or aggregation age (Fig 8C), and distribution of μNS aggregate amount at death points was almost identical to that at the termination of the measurements for the survived lineages (Fig 8D). These results suggest that, as noted for endogenous Hsp104-associated protein aggregation, induction of ectopic protein aggregation causes no functional loads on *S*. *pombe* cells in terms of the rates of cell division and initiation of death.

### Deletion of *hsp104*^+^ gene does not result in aging

**Fig 9:**
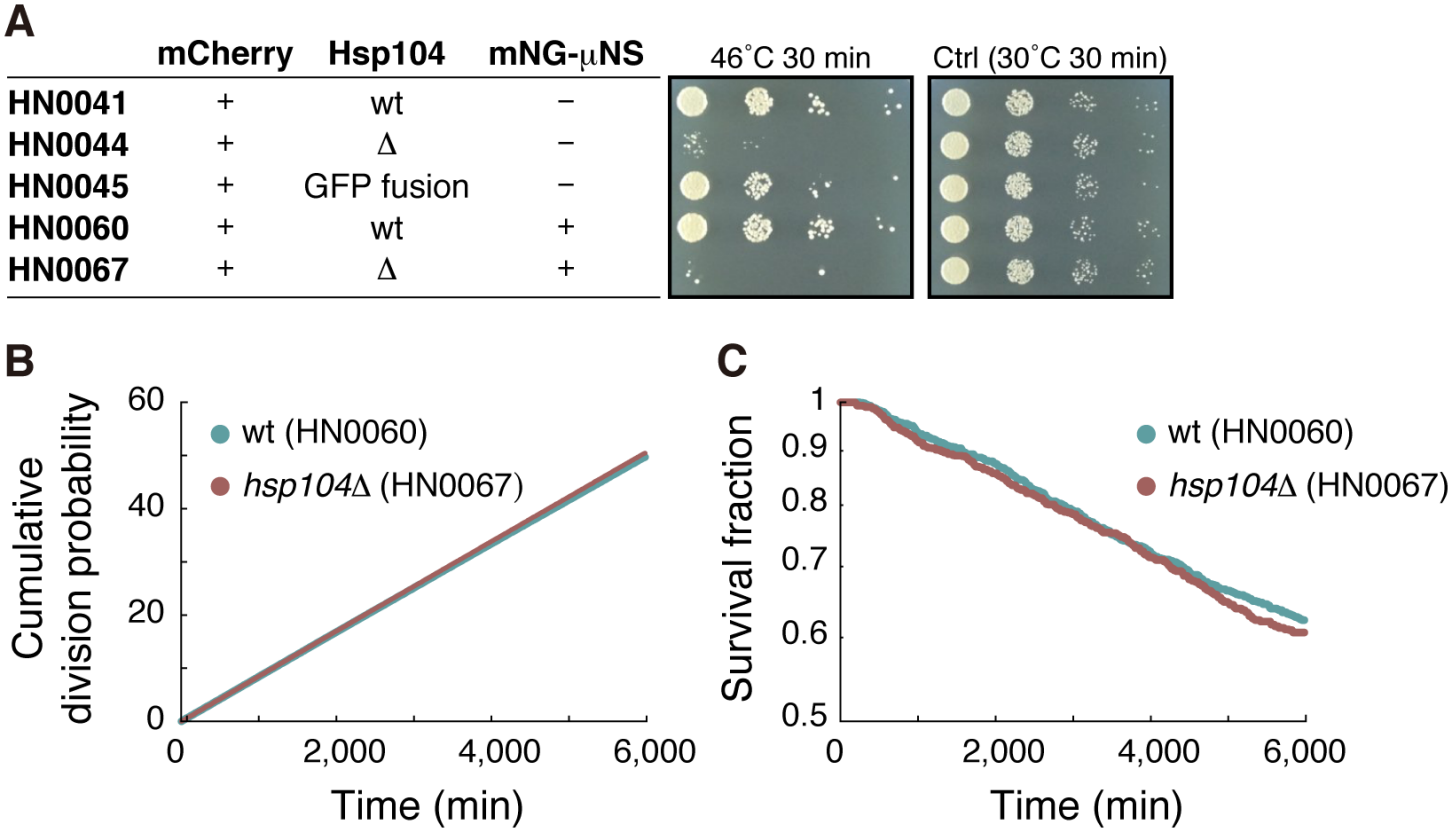
Disruption of *hsp104*^+^ does not cause replicative aging. (A) Spot assays showing that *hsp104Δ* is sensitive to heat shock. (B) The cumulative division probability plotted against time. Division rates estimated by the slope of the fitted lines are (8.319 ± 0.001) × 10^−3^ min^-1^ for wild type and (8.448 ± 0.001) × 10^−3^ min^-1^ for *hsp104Δ*. (C) Survival curves. Death rates estimated by exponential fitting are (8.55 ± 0.01) × 10^−5^ min^-1^ for wild type and (8.85 ± 0.01) × 10^−5^ min^-1^ for *hsp104Δ*. Ctrl, control; GFP, green fluorescent protein; wt, wild type. The numerical values for the plots are deposited in the Dryad repository: http://dx.doi.org/10.5061/dryad.s2t5t/15.

Finally, we examined if *hsp104*^+^ gene disruption results in replicative aging in the old-pole cell lineages. As expected, *hsp104Δ* strains were sensitive to heat shock (Fig 9A). The deletion, however, did not affect the status of μNS aggregate in terms of both inheritance duration and distribution of the amount (S8A and S8B Fig). Likewise, generation time remained noncorrelated to aggregation (S8C Fig), and distribution of aggregate amount at death points was similar to that at the end points of the survived lineages (S8D Fig). Division and death rates for the deletion mutant were essentially identical to those in the wild-type strain (Fig 9B and 9C). Taken together, our data suggest that regulation of protein aggregation by Hsp104 is not critical for avoiding replicative aging in the old-pole lineages of *S*. *pombe*.

## Discussion

In this study, we investigated the growth and death of *S*. *pombe* under various favorable culture conditions and demonstrated the lack of replicative aging in old-pole cell lineages. While being free from replicative aging, the cells died abruptly, and our extensive quantification revealed trade-offs between reproduction rate and survival probability. Furthermore, we showed that Hsp104-associated protein aggregation, often regarded as an aging factor, did not have a significant effect on growth and initiation of the dying processes, not only under normal conditions but also when stressed, where a large quantity of protein aggregate was induced.

### Replicative aging in fission yeast

The observation that both division and death rates were constant over tens of generations strongly suggested that the fission yeast old-pole cell lineages were free from replicative aging (Figs 1 and 2). This endorses the conclusion of a recent report on the absence of replicative aging in fission yeast in favorable conditions [11,27]. However, we cannot formally exclude the possibility that aging of fission yeast occurs over a longer timescale than the observed durations in our experiments and in other recent work [11,27]. Regarding the discrepancy with the earlier reports, in which replicative aging was suggested [8,10], it is of note that in the cited mortality assays, the populations became extinct within 20 generations, while about 80% of cells survived at that generation in our experiments (Fig 2E). This implies that the physiological states of the observed cells in the experiments reported elsewhere were significantly different from those in ours.

Recently, Spivey et al. have also shown the lack of replicative aging in old-pole cell lineages of *S*. *pombe* under favorable conditions using a similar microfluidics device [27]. Importantly, they observed that the death rates were variable among different strains of *S*. *pombe* even under the same culture conditions. For example, the death rate of NCYC132 strain was 5-fold lower than that of *h*^-^ *972*. On the other hand, we have shown that the death rates of one *S*. *pombe* strain (*h*^-^ *972*) are variable under different growth conditions and characterized the trade-off between fast growth and death (Fig 3). It is then natural to ask how strongly the division-death trade-off depends on the genetic backgrounds of different strains. This important subject of future studies might provide insights into the generality and the origin of the trade-off relation.

Our experiments involving oxidative stress exposure in the microfluidic device revealed that generation time and death rate reverted to normal after removal of the stressor, although most old-pole cells continued to inherit and accumulate even more Hsp104-associated protein aggregates (Fig 7). This result is inconsistent with the previous report [11], and the widely accepted notion that *S*. *pombe* ages under stressful conditions. Because oxidative stress conditions used in our study (1-h exposure to 2 mM H_2_O_2_) are more severe than those in [11] (1-h exposure to 1 mM H_2_O_2_), the absence of noticeable aging phenotypes cannot be explained by an insufficient dose of stress. One of the possible causes of the discrepancy might be the difference in the culture conditions after stress treatment: cells were grown in microcolony on an agarose pad in [11], whereas they were grown under a microfluidic device with a continuous liquid medium supply in our study. The lack of progressive increases in generation time suggests that the observed increases of generation time, death rate, and the amounts of Hsp104-associated protein aggregation are responses provoked by the stress exposure, not the signature of aging. Therefore, aging under stressful conditions might not be a general trait in *S*. *pombe*.

The lack of noticeable aging in *S*. *pombe* old-pole lineages contrasts with that in *E*. *coli* cultured under favorable growth conditions in the Mother Machine, in which division rates of old-pole cells were stable, but death rates increased over generations [7]. It is interesting to note that the modes of cell wall syntheses at poles are quite different between the 2 symmetrically dividing microorganisms. *S*. *pombe* employs polar growth, and newly synthesized cell wall materials are exclusively incorporated at the ends of the cells [35–37], whereas in *E*. *coli*, the cylindrical part of the cell grows and cell walls at poles are thought to be metabolically inert and unable to avoid deterioration [38–40]. Thus, for fission yeast we do not have an a priori reason to believe that old-pole lineages should undergo senescence. Rather, new-pole lineages that inherit a larger proportion of old lateral cell walls and a birth scar (due to the delay for the new-pole end to initiate growth after division [41]) might be subject to aging. It should be stressed that our results do not rule out the existence of any forms of lineage-specific aging in fission yeast, e.g., lineages that inherit damaged (carbonylated) proteins and/or birth scars [10]. Since the Mother Machine allows tracking of only old-pole cell lineages, new methods are required to track such cell lineages within proliferating populations.

Our results might also imply that symmetrically dividing unicellular organisms could escape aging if they possess efficient damage repair mechanisms. On the other hand, systematic asymmetric partitioning of most cellular components should occur in nonsymmetrically dividing unicellular organisms, resulting in consistent unequal partitioning of the damaged materials to specific lineages, associated with cellular morphological features such as cell size. This might underlie the empirical fact that aging is more prominent in asymmetrically dividing unicellular organisms.

### Protein aggregation and cellular growth/death

A common perception is that protein aggregate accumulates during the aging process or the stress response, and cells die catastrophically when the aggregation load exceeds the cellular capacity. Our data, however, indicated that Hsp104-associated protein aggregate is also formed in aging-free cell lineages (Fig 4 and S4 Movie). We showed that neither the aggregate amount nor the retention time affected the generation time (Fig 5). In addition, we demonstrated that cells transiently exposed to oxidative stress could promptly resume normal growth, even in the presence of unusually large amounts of protein aggregate induced by such stress (Fig 7). The commonly observed correlation between protein aggregation and cell death is most likely explained by the accelerated accumulation of aggregate a few generations before cell death. The commencement points of accelerated accumulation appear to specify the initiation points of the dying processes because the other abnormalities of mCherry expression levels and cellular morphology started around the same time. The initiation of the dying process occurred irrespectively of aggregate quantity, which argues against the concept that there is an absolute threshold in protein aggregation burden for triggering cell death. The results also suggest that retention of the aggregates did not elevate the cell death probability (Fig 6H–6J). Overall, our data indicate that Hsp104 foci do not reflect gradual deterioration of proteostasis in the cells and thus cannot be used as the sole molecular marker for cellular senescence in fission yeast. Our results do not exclude the possibility, however, that rapid accumulation of protein aggregates might accelerate completion of the dying processes post onset. For example, it has been suggested that large protein aggregates tend to overlap division plane and increase the frequency of death [11]. Characterizing in more detail the intracellular events that occur concurrently with the accelerated accumulation might unravel the new roles of the protein aggregates in *S*. *pombe*.

Together with the results regarding Hsp104-associated protein aggregation, our experimental results involving the ectopic aggregation-prone protein, μNS, suggest that *S*. *pombe* is, in fact, highly tolerant of protein aggregation loads (Fig 8) and that their adverse effects on growth and death and their relation to aging might have been overestimated. More insights would be gained by examining the correlations between the other types of foreign protein aggregation and cellular division and death.

It should be noted that there can be multiple types of protein aggregation with different constituents and formation/degradation dynamics, such as stress foci/Q-bodies/CytoQ, immobile protein deposit (IPOD), juxtanuclear quality control compartment (JUNQ)/intranuclear quality control compartment (INQ), and age-associated deposits, as reported for budding yeast [17,42–44]. Hsp104 is enriched in stress foci, IPOD, and age-associated deposits, but typically not in JUNQ/INQ [17]. Although Hsp104 is assumed to represent total protein aggregates in fission yeast [18], detailed classification and characterization of protein aggregates in this organism are still lacking. Therefore, future studies should clarify to what extent Hsp104-associated protein aggregates capture the behaviors of total protein aggregates in cells.

### Trade-off between reproduction and survival

We found that as the cell division rate elevated, the death rate increased in a linear fashion (Fig 3A). Although a simple extrapolation of the linear trend predicts immortality of single cells when the division rate is below *r*^min^, we have not been able to experimentally achieve stable growth with such a low division rate under our current measurement setup. The slow growth of cells with a division rate close to, or even smaller than, *r*^min^ could be achieved by applying stressors, such as high/low temperatures, nutrient limitation, drug exposure, or harsh chemical conditions (e.g., extreme redox environments and high/low osmolarity). However, stress responses would render internal cellular states different from those in nonstressed conditions. We speculate that this linear trend is a hallmark of a balanced growth state, rather than a universal constraint on cell division and death rates in any environment.

Why might death rate be positively correlated with division rate? In line with the historical free radical theory of aging, it could be contemplated that faster growth with higher metabolic rates generates greater oxidative stress and/or toxic metabolic wastes that result in cell death [45]. Although a substantial body of work supports the notion that reactive oxygen species (ROS) underlie aging and/or the reproduction-survival trade-off, skeptical views have also been proposed in recent studies [46–48]. In addition, caution should be exercised when applying these theories to aging and/or life history, given the lack of aging before death in our observations. Another, and not mutually exclusive, possibility is that cells might allocate energy and resources to growth and division at the expense of maintenance mechanisms such as DNA repair, protein quality control, and stress responses. Indeed, the expression of stress-induced genes is negatively correlated with growth rate in budding yeast [49], and enhanced stress resistance in slower growing cells has been reported [50,51]. Demetrius proposed that it is neither metabolic rate nor specific metabolites, but rather the stability of the entire metabolic system that determines lifespan [52]. However, how external environments (i.e., temperature and/or nutrition status) affect the robustness of steady-state levels of metabolites to stochastic fluctuations in metabolic processes is still poorly understood and would be an interesting topic for future research.

### Causes of cell death

In general, death can be triggered by both external and internal cues [53]. Because the microfluidic system ensures stable culture conditions by continuous supply of fresh medium, cellular deaths observed in this study are most likely caused by internal signals, although we cannot exclude the possibility that subtle fluctuations of local environments around cells had some impacts on death. DNA lesions are unlikely to be a major cause of death because the extensive elongation in cell length, a characteristic phenotype of DNA damage response [54], was observed in only approximately 20% of cases (S4E Fig). ROS are involved in many examples of programmed cell death from yeasts to humans [55–57] and thus are at least one of the significant candidates to be examined. Accordingly, it would be of great interest to visualize the dynamics of mitochondria and/or peroxisomes in our experimental system. A very recent study has reported that Sir2p (silent information regulator 2: a highly conserved NAD^+^-dependent deacetylase) overexpression or inhibition of the TOR pathway (target of rapamycin: a highly conserved phosphatidylinositol kinase-related protein kinase that is responsible for nutrient responsive growth regulation) by rapamycin decreases death rates in *S*. *pombe* [27]. Involvement of a variety of biological processes implicated by such observations, including heterochromatin regulation [58,59], asymmetric partition of damaged proteins [10], and nitrogen-starvation responses [60–62], should be investigated in future research.

At the present time, however, we do not understand if sporadic death is dependent on any specific molecular mechanism. For example, intrinsic fluctuations in gene expression and/or biochemical reaction rates may well differentially result in catastrophic alterations to cellular physiology. A variety of fluorescent biosensors for more global physiological states, such as intracellular pH, ATP, NAD^+^/NADH, molecular crowding, and second messengers—cAMP, diacylglycerol (DAG), Ca^++^, etc.—would be valuable and informative to infer the causes of cell death [63–68]. We hope that our simple but powerful microfluidics approach can contribute to the detailed study of the mechanisms of cell mortality and a deeper understanding of limitations in cellular homeostasis, one of the significant questions in biology.

## Materials and methods

### Microfluidic device fabrication

The microfluidic device was fabricated by standard photolithography techniques (S1B Fig) [69–71]. The CAD designs, created using ZunoRAPID software (Photron), were printed on photoresist-coated chrome-on-glass masks (CBL4006Du-AZP, Clean Surface Technology) using a laser-drawing system (DDB-201-TW, Neoark). The UV-exposed regions of the photoresist (AZP1350) were removed by NMD-3 (Tokyo Ohka Kogyo), and the exposed chromium was etched by MPM-E350 (DNP Fine Chemicals). After removing the remaining photoresist layer using acetone (Wako), the masks were rinsed with MilliQ water and air-dried.

An SU-8 mold for the PDMS device was made on a silicon wafer (ϕ = 76 mm, P<100>, resistance 1 to 10 Ω·cm, thickness 380 μm; Furuuchi Chemical) in 2 steps. First, to make observation channels, the wafer was coated with SU-8 3005 (Nippon Kayaku) using a spin-coater (MS-A150, Mikasa) at 500 rpm for 10 s and then at 4,000 rpm for 30 s. After soft baking for 2 min at 95°C, the photoresist was exposed to UV using the mercury lamp of a mask-aligner (MA-20, Mikasa) at 22.4 mW/cm^2^ for 12 s. Postexposure baking was performed at 95°C for 3 min, followed by exposure to SU-8 developer (Nippon Kayaku) and a 2-propanol (Wako) rinse. The same procedure was repeated to fabricate trenches using SU-8 3025 (Nippon Kayaku) at 500 rpm for 10 s and then at 2,000 rpm for 30 s. Soft baking was performed at 95°C for 10 min, followed by UV exposure at 22.4 mW/cm^2^ for 16 s and postexposure baking at 95°C for 10 min.

The PDMS base and curing agent (Sylgard 184) were mixed at a ratio of 10:1, poured onto the SU-8 mold in a container, and degassed using a vacuum desiccator. Curing was performed at 65°C overnight. The device was peeled from the mold, washed briefly in ethanol with sonication, and then air-dried. After punching 2 holes in the device to connect the inlet and outlet tubes, the surfaces of the device and a coverslip (24 × 60 mm, thickness 0.12–0.17 mm, Matsumani) were activated using a plasma cleaner (PDC-32G, Harrick Plasma) and bonded together. Finally, the inlet and outlet tubes were inserted into the holes (see also S1 Fig).

### Fission yeast strains

HN0025, which expresses mVenus under the control of a constitutive *adh1* promoter (*h*^-^ *leu1-32*::*leu1*^+^*-Padh1-mVenus*), was constructed by transforming HN0003 (*h*^-^ *leu1-32*) with the *Not* I-digested fragment of pDUAL-mVenus, generated by replacing the *Sph* I-*Cla* I fragment of pDUAL 13G10 with the *Padh1-mVenus-nmt1 terminator* cassette. HN0041 was constructed in the same manner as HN0025, except that the mVenus fragment was replaced with mCherry, and used as a parental strain to establish HN0045. To generate HN0045, the GFP tag was fused at the C-terminus of Hsp104 by PCR-based gene targeting [72]: the 3′ end of the ORF and 3′ UTR region of *hsp104*^+^ were assembled with the GFP(S65T)-*kan*^*R*^ fragment on the pFA6a-GFP(S65T)-KanMX6 to generate a targeting module. For the strain depicted in S1D Fig (HN0034), the *Padh1-mCherry* cassette was integrated at the *ade6*^+^ locus. To generate HN0060, *Ptef-mNeonGreen-μNS* cassette was first cloned into pDUAL vector, and then a *Not* I-digested fragment was integrated to the *leu1-32* locus. Note that the μNS is a truncated version (1,411–2,163) that corresponds to amino acids 471–721. Genomic DNA of bacterial strain CJW4617 [34] (a kind gift from Dr. M. Nibert at Harvard Medical School and Dr. C. J. Wagner at Yale University) was used as a template for PCR cloning of the μNS cDNA. To disrupt *hsp104*^+^, the host strains were transformed by a PCR-amplified deletion cassette, and G418-resistant clones were selected. A complete list of the strains and PCR primers used in this study can be found in S4 Table. The pDUAL 13G10 strain was provided by Riken BRC, a member of the National Bio-Resources Project of the MEXT, Japan [73,74].

### Confocal microscopy

Confocal fluorescence microscopic images of yeast cells in the microfluidic device (S1D Fig) were acquired using a Nikon Ti-E microscope equipped with a laser scanning confocal system, equipped with a 60× objective lens (Plan Apo λ N.A. 1.4, Nikon) and under oil immersion. The resolution along the Z-axis was 0.15 μm, and 335 images (corresponding to approximately 50 μm in height) were taken. Three-dimensional reconstructions of the images were achieved using an ImageJ 3D viewer plug-in.

### Long-term time-lapse experiments

For long-term time-lapse measurements, 10 mL of a log-phase culture of yeast cells at 28–34°C in YE containing 3% glucose or EMM containing 2% glucose was concentrated 50-fold by centrifugation and injected into the microfluidic device using a 1 mL syringe (Terumo). Cells were loaded into the observation channels by gravity, simply slanting the device. The loading procedure typically took a couple of hours, during which time the cells often entered into an early stationary phase in response to the highly crowded environment, resulting in a time lag before stable growth was achieved. The device was supplied with appropriate medium supplemented with a low concentration (10 μg/mL) of ampicillin sodium (Wako) to minimize the risk of bacterial contamination. Note that ampicillin has no effect on fission yeast growth. The flow rate was 10–15 mL/h. For transient oxidative stress treatment, the medium was changed to YE containing 2 mM hydrogen peroxide for 1 h and then switched back to YE.

We used a Nikon Ti-E microscope with a thermostat chamber (TIZHB, Tokai Hit), 40× objective (Plan Apo λ N.A. 0.95, Nikon), cooled CCD camera (ORCA-R2, Hamamatsu Photonics), and an LED excitation light source (DC2100, Thorlabs). Several cell divisions were allowed before initiating measurements. Micromanager software (https://micro-manager.org/) was used for fluorescence and/or bright field image acquisition. The time-lapse interval was 3 min (for the experiments described in Figs 1–3) or 5 min (for the experiments described in Figs 4–9). Exposure times were 400 ms (for mVenus), 200 ms (for GFP), 100 ms (for mCherry), and 10 ms (for bright field).

### Single-cell lineage tracking and division/death point identification

The acquired fluorescence images were converted into binary images using a custom-written OpenCV program. The binary images were used to identify cellular regions or ROIs, and lineage tracking (relating ROIs along lineages) was performed using a customized ImageJ macro. The transition between ROIs along each lineage was analyzed to mark cell division points where an ROI area suddenly decreased more than 1.5-fold. To mark cell death points, 2 criteria were employed: (1) if there was no division during a 360-min window, then the beginning of the window was defined as a death point, and (2) if there was a profound (more than 1.75-fold) decrease in fluorescence during a 30-min time window, then the beginning of the window was defined as a death point. We confirmed that the decay curve of surviving cell lineages obtained using these death criteria quantitatively concurred with that obtained by manual image inspection (S4A Fig). In the data set used in Figs 6–9, we examined all of the cell-size trajectories by eye and manually marked death points so as to ensure confidence in the data.

The death onset points (= kinks on the aggregate amount trajectories) were identified by manually inspecting the aggregate amount trajectory plots for all of the 541 extinct lineages. Aggregate amount/age at the kinks and generations to die after the onset of the dying process were subsequently recorded using a custom-developed ImageJ-plugin.

Lineage tracking data for all the environments tested are deposited in the Dryad repository [75].

### Estimation of the population doubling time from the distribution of intrinsic generation time

Generation time distributions obtained by following old-pole cell lineages in the Mother Machine represent intrinsic cellular division properties when 2 sister cells are physiologically indistinguishable. In standard batch cultures, however, selection occurs because of heterogeneities in cellular generation times, which cause the population doubling time (*T*_*d*_) to be smaller than the mean generation time ${\langle \tau \rangle }_{g}=\int_{0}^{\infty }\tau g\left(\tau \right)d\tau$. Here *g*(*τ*) is the probability density function of an intrinsic generation time distribution in a given culture condition. When cells randomly and independently determine their generation times according to *g*(*τ*), the population growth rate, Λ = ln2/*T*_*d*_, must satisfy the Euler-Lotka equation [28–30],

$$1=\int_{0}^{\infty }2e^{-\Lambda \tau }g\left(\tau \right)d\tau .$$ (1)

Equivalently, (Eq 1) can be rewritten as

$$1=2\Lambda \int_{0}^{\infty }e^{-\Lambda \tau }B\left(\tau \right)d\tau ,$$ (2)

where $B\left(\tau \right)\equiv \int_{\tau }^{\infty }g\left(\tau^{\prime }\right)d\tau^{\prime }$ is the survival function (complementary cumulative distribution) of *g*(*τ*), which represents the probability of a newly divided cell remaining undivided until age *τ*. One can show that ${\langle \tau \rangle }_{g}=\int_{0}^{\infty }B\left(\tau \right)d\tau$, and then $\psi_{l}\left(\tau \right)=B\left(\tau \right)/{\langle \tau \rangle }_{g},$, becomes a probability density function and can be interpreted as “age distribution” along cell lineages. Therefore, Eq 2 can be also expressed as

$$1=2\Lambda {\langle \tau \rangle }_{g}\int_{0}^{\infty }e^{-\Lambda \tau }\psi_{l}\left(\tau \right)d\tau .$$ (3)

We numerically estimate Λ based on a discretized version of Eq 3, i.e.,

$$1=2\Lambda {\langle \tau \rangle }_{g}\sum_{i}e^{-\Lambda \left(i\Delta \tau +\frac{\Delta \tau }{2}\right)}\psi_{i}\left(i\Delta \tau \right)\Delta \tau \;\;\;\;\;\;\left(i=0,\;\;1,\;\;2\;\cdots \right),$$ (4)

where Δ*τ* is the time-lapse interval. 〈*τ*〉_*g*_ was calculated as ${\langle \tau \rangle }_{g}=\frac{1}{n}\sum_{i=1}^{n}\tau_{i}$, where *τ*_*i*_ is the generation time and *n* is the number of samples, and the probability distribution of age is calculated as $\psi_{l}\left(a\right)\Delta \tau =\frac{\#\text{ of cells with age}=a}{\#\text{ of total cells}}$.

### Division rate, death rate, and expected life span of fission yeast

We calculated division rate *r* as

$$r=\langle \frac{1}{\tau }\rangle =\frac{1}{n}\sum_{i=1}^{n}\frac{1}{\tau_{i}},$$

where *τ*_*i*_ is the generation time and *n* is the number of samples.

We estimated death rate *k* from the decay curve by the least squares fitting (*t* versus ln(*N*(*t*)), where *t* is time and *N*(*t*) is the number of surviving lineages at *t*). The expected value of “time to death” is thus $\frac{1}{k}$. We calculated “expected life span” in units of generation as $\frac{r}{k}$.

### Error estimation of division and death rates

The standard errors for the division rates were calculated as

$$S.E._{\text{division}}=\frac{\sigma_{\text{division}}}{\sqrt{n}}=\sqrt{\frac{\frac{1}{n}\sum_{i=1}^{n}{\left(\frac{1}{\tau_{i}}\right)}^{2}-{\left(\frac{1}{n}\sum_{i=1}^{n}\frac{1}{\tau_{i}}\right)}^{2}}{n}},$$

where *τ*_*i*_ is the generation time and *n* is the number of samples. ±2*S*. *E*._division_ ranges were shown as error bars in Fig 3A and 3B.

To evaluate errors in the death rate estimations, we produced simulated decay curves of the surviving fraction using parameters (death rate, initial cell number, and observation period) specific to each experiment. The simulation was repeated 5,000 times for each environment, and the death rate was obtained from a simulated survival curve in each run. The standard deviation of the determined death rates was calculated as *σ*_death_, and the ±2*σ*_death_ ranges were shown as error bars in Fig 3A. Errors in expected life span (*σ*_lifespan_) were calculated using the error propagation rule.

### Statistical evaluation of death (or death onset) probability

We first estimated death probability per generation *p*_*0*_ to be 1.15 × 10^−2^ from the survival curve. In Fig 6F and 6I, the death probability *p* for each aggregate amount or aggregation age was then tested using a binomial test for the two-tailed null hypothesis *H*_*0*_: *p = p*_*0*_ at the significance level = 0.05.

For the onset probability of accelerated accumulation, we set the null hypothesis to be *H*_*0*_: *q* = 0.79 *p*_*0*_ = 9.09 × 10^−3^ based on our observation that a clear kink in the protein aggregation dynamics was detected in 79% of the extinct lineages and implemented binomial testing at the significance level = 0.05 (Fig 6G and 6J).

## Supporting information

S1 Table Summary of measurements. (DOCX)

S2 Table Comparison of cellular growth between batch culture and the microfluidic device. Population doubling times estimated by growth curves of batch cultures are shown with standard errors. Mean generation time was calculated as the arithmetic mean. Uncertainty comes from the time resolution (3 min in these cases) of measurements. Population doubling time is generally shorter than mean generation time, and can be estimated using the Euler-Lotka equation (see Methods).
(DOCX)

S3 Table Summary of division and death rate estimations. These data were used for the plots shown in Fig 3.
(DOCX)

S4 Table List of fission yeast strains. All the strains used in this study were constructed by crossing Bern collection K strains (*L972 h*^-^ and *L975 h*^+^ derivatives).
(DOCX)

S1 Fig Fabrication procedures and setup of the PDMS device. (A) Microscopic images of a SU-8 mold on a silicon wafer. (Top) An image showing three trenches and four observation channel arrays. (Bottom) An enlarged view of one observation channel array. (B) Fabrication and assembly procedures for the PDMS microfluidic device. (C) A illustration of the microfluidic device connected to silicon tubing and a sample loading syringe. (D) A 3D view of the device and loaded cells reconstructed from confocal microscopic images. Cells expressing mCherry were delivered into the device with medium containing 3 μM fluorescein. Green fluorescence (left) and red fluorescence (right) images are presented. A 3D reconstruction was achieved using an ImageJ 3D viewer plug-in.
(PDF)

S2 Fig Medium exchanges in the microfluidic device. (A) Time-lapse fluorescence images showing fluorescein removal. The device was first filled with YE containing 3 μM fluorescein, and then YE medium without fluorescein was supplied at a flow rate of 10 mL/h. (B) Fluorescein removal at different flow rates. The same experiments as in (A) were performed at various flow rates, and the decay of fluorescence intensity was plotted against time (left). Fluorescence intensity was normalized to the values at *t* = 0. The points indicate the decay of fluorescence in the observation channels, and the lines indicate this decay in trenches. The 90% decay time was less than 5 min when the flow rate was greater than 10 mL/h (right). The experiments described in the main text were performed at 10–15 mL/h. (C) Quick introduction of fluorescent dye into observation channels. After loading of cells, YE medium containing 20 μg/mL of Calcofluor White Stain (Sigma-Aldrich), which stains cell walls, especially septa, was supplied at a flow rate of 10 mL/h. Cells in both narrow and wide observation channels were stained with the same kinetics, suggesting that the medium was effectively supplied even in the presence of cells in the thin observation channels. It is also of note that the cells at the ends of the channels were stained as efficiently as those at the exits of the channels.
(PDF)

S3 Fig Cumulative division probability for all tested environments. Linear fitting was performed using the time window after the gray vertical lines, where stable cellular growth was achieved.
(PDF)

S4 Fig Characterization of the spontaneous cell death of *S. pombe*. (A) Comparison between automated and manual estimations of death rates. To validate the estimations of death rates based on our automated cell death detection algorithm, the survival curves were compared with those obtained by manual inspections. For manual estimations of death rates, surviving old-pole cells were counted by eye every 1,000 min at all positions, and the exponential decay rates were estimated by least squares fitting. Although the automated algorithm might overestimate death rates, the differences between the automated and manual estimations were minor (at most 10%), and the survival curves were similar. (B) Photodamage effects on cell deaths. To evaluate photodamage induced by the excitation light used in fluorescence imaging, a long-term time-lapse experiment was performed with only bright-field imaging in YE at 30°C. The surviving fraction was plotted against time (light blue open circles) and compared with that in fluorescence imaging (red closed circles) in the same medium and temperature. The estimated death rate in fluorescence imaging was 20% higher (8.35/6.94 = 1.20) than that in bright-field imaging. (C) Summary of the estimated death rates in (A) and (B). (D) Classification of the death modes. Cells exhibited three types of morphological changes before death: swollen, hyper-elongated, and shrunken. Examples of bright-field or fluorescence microscopic images of these three types are shown. Dead/dying cells are indicated by arrowheads. Scale bars indicate 10 μm. (E) Fraction of each death mode in different environments. Approximately 80% of spontaneous cell deaths are classified as Type I (swollen and synchronous) in all the tested environments. (F) Synchronous cell deaths in other microfluidic device. The device has the same architecture as the Mother Machine-type device described in the main text, except that the observation channels are wider and can accommodate more cells. Progenies of a common ancestor cell (indicated by a magenta circle at t = 0 min) synchronously began to show aberrant morphological changes (t = 450 min). The surrounding cells, however, maintained normal growth characteristics. Scale bars indicate 10 μm. (G) A model of a course of spontaneous cell death in *S*. *pombe*. After receiving unknown death trigger(s), cells typically undergo a small number of divisions, during which Hsp104-associated protein aggregation is promoted.
(PDF)

S5 Fig No replicative aging in the old-pole cell lineages. Mean generation times for each generation are plotted with error bars representing standard deviations.
(PDF)

S6 Fig Survival curves for all tested environments. The surviving fractions of the old-pole cell lineages were plotted against time in a semi-log plot. Death rates were stable in the time windows after the gray vertical lines, where stable growth was achieved (see S3 Fig).
(PDF)

S7 Fig Quantitative measurement of protein aggregation. (A) An algorithm of identification of protein aggregate foci. (B) An example image of identified aggregate foci. The top image shows the representative Hsp104-GFP fluorescence, and the bottom image illustrates the identified aggregates outlined in yellow. (C) Representative images of cells with different amounts of aggregate.
(PDF)

S8 Fig Deletion of *hsp104*^*+*^ does not affect protein aggregation status. (A) Distributions of inheritance duration of mNeonGreen-μNS aggregate. (B) Distributions of aggregate amount of mNeonGreen-μNS. (C) Density plots showing the relations between generation time and aggregate amount (left) and between generation time and aggregation age (right). The plots for both wildtype and hsp104Δ strain are presented. (D) Distributions of mNeonGreen-μNS aggregate amounts at death points (red) and at the end of the measurements for the surviving lineages (blue). The left plot shows the result for wildtype; and the right plot for hsp104Δ strain.
(PDF)

S1 Movie Medium is rapidly exchanged in the microfluidic device. (Top left) The device was first filled with YE medium, and then YE medium containing fluorescein was supplied at a flow rate of 10 mL/h. The time-lapse interval was 15 sec. (Bottom) Medium components can reach the ends of the observation channels. YE medium containing Calcofluor White, which stains cell walls and septa, was supplied at a flow rate of 10 mL/h. (Bottom left) Bright field images. (Bottom right) Fluorescence images of the Calcofluor-stained cells. The time-lapse interval was 15 sec.
(MOV)

S2 Movie Typical time-lapse images and conversion to binary images. Time-lapse movie of strain HN0025 cultured in the microfluidic device in YE at 28°C (left), and corresponding binarized mask images (right). The time-lapse imaging interval was 3 min.
(MOV)

S3 Movie Synchronous cell death. Time-lapse movie of strain HN0045 cultured in YE at 32°C. The PDMS microfluidic device has wider observation channels than the Mother Machine described in the main text. The progenies of a single common ancestor cell (indicated by yellow circles at the beginning of the movie) died synchronously without affecting growth of the surrounding cells.
(MOV)

S4 Movie Dynamics of protein aggregation and clearance. Time-lapse movie of strain HN0045 cultured in the microfluidic device in YE at 32°C. Two sets (GFP channel for Hsp104-GFP and RFP channel for mCherry) of fluorescence images were merged. The time-lapse imaging interval was 5 min, and images captured every 10 min were used to assemble the movie. Green: Hsp104-GFP. Magenta: mCherry.
(MOV)

S5 Movie Dynamics of μNS aggregation and segregation. Time-lapse movie of strain HN0060 cultured in the microfluidic device in YE at 32°C. Two sets (YFP channel for mNeonGreen-μNS and RFP channel for mCherry) of fluorescence images were merged. The time-lapse imaging interval was 5 min, and images captured every 10 min were used to assemble the movie. Green: mNeonGreen-μNS. Magenta: mCherry.
(MOV)

## Abbreviations

ctrl: control
DAG: diacylglycerol
EMM: Edinburgh minimal medium
GFP: green fluorescent protein
INQ: intranuclear quality control compartment
IPOD: immobile protein deposit
JUNQ: juxtanuclear quality control compartment
PDMS: polydimethylsiloxane
RFP: red fluorescent protein
ROI: region of interest
ROS: reactive oxygen species
Sir2p: silent information regulator 2
TOR: target of rapamycin
wt: wild type
YE: yeast extract medium

## References

[1] Ackermann M. Senescence in a Bacterium with Asymmetric Division. Science (80-). 2003;300: 1920 doi: 10.1126/science.1083532 12817142 10.1126/science.1083532

[2] Jazwinski SM. The genetics of aging in the yeast Saccharomyces cerevisiae. Genetica. 1993;91: 35–51. doi: 10.1007/BF01435986 8125278 10.1007/BF01435986

[3] Fu X, Meng F, Hu Y, Zhou J. Candida albicans, a distinctive fungal model for cellular aging study. Aging Cell. 2008;7: 746–57. doi: 10.1111/j.1474-9726.2008.00424.x 18691183 10.1111/j.1474-9726.2008.00424.x PMC2773528

[4] Stewart EJ, Madden R, Paul G, Taddei F. Aging and death in an organism that reproduces by morphologically symmetric division. PLoS Biol. 2005;3: e45 doi: 10.1371/journal.pbio.0030045 15685293 10.1371/journal.pbio.0030045 PMC546039

[5] Lindner AB, Madden R, Demarez A, Stewart EJ, Taddei F. Asymmetric segregation of protein aggregates is associated with cellular aging and rejuvenation. Proc Natl Acad Sci U S A. 2008;105: 3076–81. doi: 10.1073/pnas.0708931105 18287048 10.1073/pnas.0708931105 PMC2268587

[6] Winkler J, Seybert A, König L, Pruggnaller S, Haselmann U, Sourjik V, et al Quantitative and spatio-temporal features of protein aggregation in Escherichia coli and consequences on protein quality control and cellular ageing. EMBO J. 2010;29: 910–23. doi: 10.1038/emboj.2009.412 20094032 10.1038/emboj.2009.412 PMC2837176

[7] Wang P, Robert L, Pelletier J, Dang WL, Taddei F, Wright A, et al Robust growth of Escherichia coli. Curr Biol. 2010;20: 1099–103. doi: 10.1016/j.cub.2010.04.045 20537537 10.1016/j.cub.2010.04.045 PMC2902570

[8] Barker MG, Walmsley RM. Replicative ageing in the fission yeast Schizosaccharomyces pombe. Yeast. 1999;15: 1511–8. doi: 10.1002/(SICI)1097-0061(199910)15:14<1511::AID-YEA482>3.0.CO;2-Y 10514568 10.1002/(sici)1097-0061(199910)15:14<1511::aid-yea482>3.3.co;2-p

[9] Minois N, Frajnt M, Dölling M, Lagona F, Schmid M, Küchenhoff H, et al Symmetrically dividing cells of the fission yeast schizosaccharomyces pombe do age. Biogerontology. 2006;7: 261–7. doi: 10.1007/s10522-006-9025-y 16821114 10.1007/s10522-006-9025-y

[10] Erjavec N, Cvijovic M, Klipp E, Nystrom T. Selective benefits of damage partitioning in unicellular systems and its effects on aging. Proc Natl Acad Sci U S A. 2008;105: 18764–9. doi: 10.1073/pnas.0804550105 19020097 10.1073/pnas.0804550105 PMC2596250

[11] Coelho M, Dereli A, Haese A, Kühn S, Malinovska L, DeSantis ME, et al Fission yeast does not age under favorable conditions, but does so after stress. Curr Biol. 2013;23: 1844–52. doi: 10.1016/j.cub.2013.07.084 24035542 10.1016/j.cub.2013.07.084 PMC4620659

[12] Doyle SM, Genest O, Wickner S. Protein rescue from aggregates by powerful molecular chaperone machines. Nat Rev Mol Cell Biol. 2013;14: 617–29. doi: 10.1038/nrm3660 24061228 10.1038/nrm3660

[13] Mokry DZ, Abrahão J, Ramos CHI. Disaggregases, molecular chaperones that resolubilize protein aggregates. An Acad Bras Cienc. 2015;87: 1273–92. doi: 10.1590/0001-3765201520140671 26312418 10.1590/0001-3765201520140671

[14] Erjavec N, Larsson L, Grantham J, Nyström T. Accelerated aging and failure to segregate damaged proteins in Sir2 mutants can be suppressed by overproducing the protein aggregation-remodeling factor Hsp104p. Genes Dev. 2007;21: 2410–21. doi: 10.1101/gad.439307 17908928 10.1101/gad.439307 PMC1993872

[15] Liu B, Larsson L, Caballero A, Hao X, Öling D, Grantham J, et al The Polarisome Is Required for Segregation and Retrograde Transport of Protein Aggregates. Cell. 2010;140: 257–67. doi: 10.1016/j.cell.2009.12.031 20141839 10.1016/j.cell.2009.12.031

[16] Spokoini R, Moldavski O, Nahmias Y, England JL, Schuldiner M, Kaganovich D. Confinement to organelle-associated inclusion structures mediates asymmetric inheritance of aggregated protein in budding yeast. Cell Rep. 2012;2: 738–47. doi: 10.1016/j.celrep.2012.08.024 23022486 10.1016/j.celrep.2012.08.024

[17] Saarikangas J, Barral Y. Protein aggregates are associated with replicative aging without compromising protein quality control. Elife. 2015;4 doi: 10.7554/eLife.06197 26544680 10.7554/eLife.06197 PMC4635334

[18] Coelho M, Lade SJ, Alberti S, Gross T, Tolić IM. Fusion of protein aggregates facilitates asymmetric damage segregation. PLoS Biol. 2014;12: e1001886 doi: 10.1371/journal.pbio.1001886 24936793 10.1371/journal.pbio.1001886 PMC4061010

[19] Steffen KK, Kennedy BK, Kaeberlein M. Measuring replicative life span in the budding yeast. J Vis Exp. 2009; doi: 10.3791/1209 19556967 10.3791/1209 PMC2797481

[20] Charvin G, Cross FR, Siggia ED. A microfluidic device for temporally controlled gene expression and long-term fluorescent imaging in unperturbed dividing yeast cells. PLoS ONE. 2008;3:e1468 doi: 10.1371/journal.pone.0001468 18213377 10.1371/journal.pone.0001468 PMC2194624

[21] Bennett MR, Hasty J. Microfluidic devices for measuring gene network dynamics in single cells. Nat Rev Genet. 2009;10: 628–38. doi: 10.1038/nrg2625 19668248 10.1038/nrg2625 PMC2931582

[22] Zhang Y, Luo C, Zou K, Xie Z, Brandman O, Ouyang Q, et al Single cell analysis of yeast replicative aging using a new generation of microfluidic device. PLoS ONE. 2012;7:e48275 doi: 10.1371/journal.pone.0048275 23144860 10.1371/journal.pone.0048275 PMC3493551

[23] Tian Y, Luo C, Ouyang Q. A microfluidic synchronizer for fission yeast cells. Lab Chip. 2013;13: 4071–7. doi: 10.1039/c3lc50639h 23966136 10.1039/c3lc50639h

[24] Nobs J-B, Maerkl SJ. Long-term single cell analysis of S. pombe on a microfluidic microchemostat array. PLoS ONE. 2014;9:e93466 doi: 10.1371/journal.pone.0093466 24710337 10.1371/journal.pone.0093466 PMC3977849

[25] Crane MM, Clark IBN, Bakker E, Smith S, Swain PS. A microfluidic system for studying ageing and dynamic single-cell responses in budding yeast. PLoS ONE. 2014;9:e100042 doi: 10.1371/journal.pone.0100042 24950344 10.1371/journal.pone.0100042 PMC4065030

[26] Spivey EC, Xhemalce B, Shear JB, Finkelstein IJ. 3D-Printed Microfluidic Microdissector for High-Throughput Studies of Cellular Aging. Anal Chem. 2014;86: 7406–12. doi: 10.1021/ac500893a 24992972 10.1021/ac500893a PMC4636036

[27] Spivey EC, Jones SK, Rybarski JR, Saifuddin FA, Finkelstein IJ. An aging-independent replicative lifespan in a symmetrically dividing eukaryote. Elife. eLife Sciences Publications Limited; 2017;6: e20340 doi: 10.7554/eLife.20340 28139976 10.7554/eLife.20340 PMC5332158

[28] Powell EO. Growth Rate and Generation Time of Bacteria, with Special Reference to Continuous Culture. J Gen Microbiol. 1956;15: 492–511. doi: 10.1099/00221287-15-3-492 13385433 10.1099/00221287-15-3-492

[29] Wakamoto Y, Grosberg AY, Kussell E. Optimal lineage principle for age-structured populations. Evolution. 2012;66: 115–34. doi: 10.1111/j.1558-5646.2011.01418.x 22220869 10.1111/j.1558-5646.2011.01418.x

[30] Hashimoto M, Nozoe T, Nakaoka H, Okura R, Akiyoshi S, Kaneko K, et al Noise-driven growth rate gain in clonal cellular populations. Proc Natl Acad Sci U S A. National Academy of Sciences; 2016;113: 3251–56. doi: 10.1073/pnas.1519412113 26951676 10.1073/pnas.1519412113 PMC4812751

[31] Magidson V, Khodjakov A. Circumventing photodamage in live-cell microscopy. Methods Cell Biol. 2013;114: 545–60. doi: 10.1016/B978-0-12-407761-4.00023-3 23931522 10.1016/B978-0-12-407761-4.00023-3 PMC3843244

[32] Broering TJ, Parker JSL, Joyce PL, Kim J, Nibert ML. Mammalian reovirus nonstructural protein microNS forms large inclusions and colocalizes with reovirus microtubule-associated protein micro2 in transfected cells. J Virol. American Society for Microbiology; 2002;76: 8285–97. doi: 10.1128/JVI.76.16.8285-8297.2002 10.1128/JVI.76.16.8285-8297.2002 PMC155143 12134034

[33] Broering TJ, Arnold MM, Miller CL, Hurt JA, Joyce PL, Nibert ML. Carboxyl-proximal regions of reovirus nonstructural protein muNS necessary and sufficient for forming factory-like inclusions. J Virol. American Society for Microbiology; 2005;79: 6194–206. doi: 10.1128/JVI.79.10.6194-6206.2005 10.1128/JVI.79.10.6194-6206.2005 PMC1091696 15858004

[34] Parry BR, Surovtsev IV, Cabeen MT, O’Hern CS, Dufresne ER, Jacobs-Wagner C. The bacterial cytoplasm has glass-like properties and is fluidized by metabolic activity. Cell. 2014;156: 183–94. doi: 10.1016/j.cell.2013.11.028 24361104 10.1016/j.cell.2013.11.028 PMC3956598

[35] Mitchison JM. The growth of single cells. Exp Cell Res. 1957;13: 244–62. doi: 10.1016/0014-4827(57)90005-8 13480293 10.1016/0014-4827(57)90005-8

[36] Johnson B. Autoradiographic analysis of regional cell wall growth of yeasts Schizosaccharomyces pombe. Exp Cell Res. 1965;39: 613–24. doi: 10.1016/0014-4827(65)90064-9 5838699 10.1016/0014-4827(65)90064-9

[37] May JW. Sites of cell-wall extension demonstrated by the use of fluorescent antibody. Exp Cell Res. 1962;27: 170–2. doi: 10.1016/0014-4827(62)90060-5 14471652 10.1016/0014-4827(62)90060-5

[38] Burman LG, Raichler J, Park JT. Evidence for diffuse growth of the cylindrical portion of the Escherichia coli murein sacculus. J Bacteriol. 1983;155: 983–8. Available: http://www.ncbi.nlm.nih.gov/pubmed/6350274 6350274 10.1128/jb.155.3.983-988.1983 PMC217789

[39] de Pedro MA, Quintela JC, Höltje JV, Schwarz H. Murein segregation in Escherichia coli. J Bacteriol. 1997;179: 2823–34. Available: http://www.ncbi.nlm.nih.gov/pubmed/9139895 9139895 10.1128/jb.179.9.2823-2834.1997 PMC179041

[40] Scheffers DJ, Pinho MG. Bacterial cell wall synthesis: new insights from localization studies. Microbiol Mol Biol Rev. 2005;69: 585–607. doi: 10.1128/MMBR.69.4.585-607.2005 16339737 10.1128/MMBR.69.4.585-607.2005 PMC1306805

[41] Mitchison JM, Nurse P. Growth in cell length in the fission yeast Schizosaccharomyces pombe. J Cell Sci. 1985;75: 357–76. Available: http://www.ncbi.nlm.nih.gov/pubmed/4044680 4044680 10.1242/jcs.75.1.357

[42] Arlia-Ciommo A, Piano A, Leonov A, Svistkova V, Titorenko VI. Quasi-programmed aging of budding yeast: a trade-off between programmed processes of cell proliferation, differentiation, stress response, survival and death defines yeast lifespan. Cell Cycle. 2014;13: 3336–49. doi: 10.4161/15384101.2014.965063 25485579 10.4161/15384101.2014.965063 PMC4614525

[43] Amen T, Kaganovich D. Dynamic droplets: the role of cytoplasmic inclusions in stress, function, and disease. Cell Mol Life Sci. 2015;72: 401–15. doi: 10.1007/s00018-014-1740-y 25283146 10.1007/s00018-014-1740-y PMC11113435

[44] Miller SBM, Mogk A, Bukau B. Spatially organized aggregation of misfolded proteins as cellular stress defense strategy. J Mol Biol. 2015;427: 1564–74. doi: 10.1016/j.jmb.2015.02.006 25681695 10.1016/j.jmb.2015.02.006

[45] Harman D. Aging: a theory based on free radical and radiation chemistry. J Gerontol. 1956;11: 298–300. doi: 10.1093/geronj/11.3.298 13332224 10.1093/geronj/11.3.298

[46] Frisard M, Ravussin E. Energy Metabolism and Oxidative Stress: Impact on the Metabolic Syndrome and the Aging Process. Endocrine. 2006;29: 27–32. doi: 10.1385/ENDO:29:1:27 16622290 10.1385/ENDO:29:1:27

[47] Selman C, Blount JD, Nussey DH, Speakman JR. Oxidative damage, ageing, and life-history evolution: where now? Trends Ecol Evol. 2012;27: 570–7. doi: 10.1016/j.tree.2012.06.006 22789512 10.1016/j.tree.2012.06.006

[48] Speakman JR, Blount JD, Bronikowski AM, Buffenstein R, Isaksson C, Kirkwood TBL, et al Oxidative stress and life histories: unresolved issues and current needs. Ecol Evol. 2015;5: 5745–57. doi: 10.1002/ece3.1790 26811750 10.1002/ece3.1790 PMC4717350

[49] Brauer MJ, Huttenhower C, Airoldi EM, Rosenstein R, Matese JC, Gresham D, et al Coordination of growth rate, cell cycle, stress response, and metabolic activity in yeast. Mol Biol Cell. 2008;19: 352–67. doi: 10.1091/mbc.E07-08-0779 17959824 10.1091/mbc.E07-08-0779 PMC2174172

[50] Elliott B, Futcher B. Stress resistance of yeast cells is largely independent of cell cycle phase. Yeast. 1993;9: 33–42. doi: 10.1002/yea.320090105 8442385 10.1002/yea.320090105

[51] Zakrzewska A, van Eikenhorst G, Burggraaff JEC, Vis DJ, Hoefsloot H, Delneri D, et al Genome-wide analysis of yeast stress survival and tolerance acquisition to analyze the central trade-off between growth rate and cellular robustness. Mol Biol Cell. 2011;22: 4435–46. doi: 10.1091/mbc.E10-08-0721 21965291 10.1091/mbc.E10-08-0721 PMC3216668

[52] Demetrius L. Caloric Restriction, Metabolic Rate, and Entropy. Journals Gerontol Ser A. 2004;59: B902–15. doi: 10.1093/gerona/59.9.B902 10.1093/gerona/59.9.b902 15472153

[53] Falcone C, Mazzoni C. External and internal triggers of cell death in yeast. Cell Mol Life Sci. Springer; 2016;73: 2237–50. doi: 10.1007/s00018-016-2197-y 27048816 10.1007/s00018-016-2197-y PMC4887522

[54] Furnari B, Rhind N, Russell P. Cdc25 mitotic inducer targeted by chk1 DNA damage checkpoint kinase. Science. 1997;277: 1495–7. Available: http://www.ncbi.nlm.nih.gov/pubmed/9278510 9278510 10.1126/science.277.5331.1495

[55] Farrugia G, Balzan R. Oxidative stress and programmed cell death in yeast. Front Oncol. Frontiers Media SA; 2012;2: 64 doi: 10.3389/fonc.2012.00064 22737670 10.3389/fonc.2012.00064 PMC3380282

[56] Low CP, Yang H. Programmed cell death in fission yeast Schizosaccharomyces pombe. Biochim Biophys Acta. 2008;1783: 1335–49. doi: 10.1016/j.bbamcr.2008.02.002 18328827 10.1016/j.bbamcr.2008.02.002

[57] Circu ML, Aw TY. Reactive oxygen species, cellular redox systems, and apoptosis. Free Radic Biol Med. NIH Public Access; 2010;48: 749–62. doi: 10.1016/j.freeradbiomed.2009.12.022 20045723 10.1016/j.freeradbiomed.2009.12.022 PMC2823977

[58] Shankaranarayana GD, Motamedi MR, Moazed D, Grewal SIS. Sir2 regulates histone H3 lysine 9 methylation and heterochromatin assembly in fission yeast. Curr Biol. 2003;13: 1240–6. Available: http://www.ncbi.nlm.nih.gov/pubmed/12867036 12867036 10.1016/s0960-9822(03)00489-5

[59] Alper BJ, Job G, Yadav RK, Shanker S, Lowe BR, Partridge JF. Sir2 is required for Clr4 to initiate centromeric heterochromatin assembly in fission yeast. EMBO J. 2013;32: 2321–2335. doi: 10.1038/emboj.2013.143 23771057 10.1038/emboj.2013.143 PMC3770337

[60] Uritani M, Hidaka H, Hotta Y, Ueno M, Ushimaru T, Toda T. Fission yeast Tor2 links nitrogen signals to cell proliferation and acts downstream of the Rheb GTPase. Genes to Cells. 2006;11: 1367–1379. doi: 10.1111/j.1365-2443.2006.01025.x 17121544 10.1111/j.1365-2443.2006.01025.x

[61] Alvarez B, Moreno S. Fission yeast Tor2 promotes cell growth and represses cell differentiation. J Cell Sci. 2006;119: 4475–4485. doi: 10.1242/jcs.03241 17046992 10.1242/jcs.03241

[62] Otsubo Y, Yamamato M. TOR Signaling in Fission Yeast. Crit Rev Biochem Mol Biol. Taylor & Francis; 2008;43: 277–283. doi: 10.1080/10409230802254911 18756382 10.1080/10409230802254911

[63] Orij R, Postmus J, Ter Beek A, Brul S, Smits GJ. In vivo measurement of cytosolic and mitochondrial pH using a pH-sensitive GFP derivative in Saccharomyces cerevisiae reveals a relation between intracellular pH and growth. Microbiology. 2009;155: 268–78. doi: 10.1099/mic.0.022038-0 19118367 10.1099/mic.0.022038-0

[64] Yaginuma H, Kawai S, Tabata K V, Tomiyama K, Kakizuka A, Komatsuzaki T, et al Diversity in ATP concentrations in a single bacterial cell population revealed by quantitative single-cell imaging. Sci Rep. Nature Publishing Group; 2014;4: 6522 doi: 10.1038/srep06522 25283467 10.1038/srep06522 PMC4185378

[65] Zhao Y, Hu Q, Cheng F, Su N, Wang A, Zou Y, et al SoNar, a Highly Responsive NAD+/NADH Sensor, Allows High-Throughput Metabolic Screening of Anti-tumor Agents. Cell Metab. NIH Public Access; 2015;21: 777–89. doi: 10.1016/j.cmet.2015.04.009 25955212 10.1016/j.cmet.2015.04.009 PMC4427571

[66] Boersma AJ, Zuhorn IS, Poolman B. A sensor for quantification of macromolecular crowding in living cells. Nat Methods. Nature Publishing Group, a division of Macmillan Publishers Limited. All Rights Reserved; 2015;12: 227–229. doi: 10.1038/nmeth.3257 25643150 10.1038/nmeth.3257

[67] Tewson PH, Martinka S, Shaner NC, Hughes TE, Quinn AM. New DAG and cAMP Sensors Optimized for Live-Cell Assays in Automated Laboratories. J Biomol Screen. SAGE Publications; 2016;21: 298–305. doi: 10.1177/1087057115618608 26657040 10.1177/1087057115618608 PMC4766961

[68] Tsien RY, Miyawaki A, Llopis J, Heim R, McCaffery JM, Adams JA, et al Fluorescent indicators for Ca^2+^ based on green fluorescent proteins and calmodulin. Nature. Nature Publishing Group; 1997;388: 882–887. doi: 10.1038/42264 9278050 10.1038/42264

[69] Quake SR. From Micro- to Nanofabrication with Soft Materials. Science (80-). 2000;290: 1536–40. doi: 10.1126/science.290.5496.1536 10.1126/science.290.5496.1536 11090344

[70] Friend J, Yeo L. Fabrication of microfluidic devices using polydimethylsiloxane. Biomicrofluidics. 2010;4 doi: 10.1063/1.3259624 20697575 10.1063/1.3259624 PMC2917889

[71] Qin D, Xia Y, Whitesides GM. Soft lithography for micro- and nanoscale patterning. Nat Protoc. 2010;5: 491–502. doi: 10.1038/nprot.2009.234 20203666 10.1038/nprot.2009.234

[72] Bähler J, Wu JQ, Longtine MS, Shah NG, McKenzie A, Steever AB, et al Heterologous modules for efficient and versatile PCR-based gene targeting in Schizosaccharomyces pombe. Yeast. 1998;14: 943–51. doi: 10.1002/(SICI)1097-0061(199807)14:10<943::AID-YEA292>3.0.CO;2-Y 9717240 10.1002/(SICI)1097-0061(199807)14:10<943::AID-YEA292>3.0.CO;2-Y

[73] Matsuyama A, Shirai A, Yashiroda Y, Kamata A, Horinouchi S, Yoshida M. pDUAL, a multipurpose, multicopy vector capable of chromosomal integration in fission yeast. Yeast. 2004;21: 1289–1305. doi: 10.1002/yea.1181 15546162 10.1002/yea.1181

[74] Matsuyama A, Arai R, Yashiroda Y, Shirai A, Kamata A, Sekido S, et al ORFeome cloning and global analysis of protein localization in the fission yeast Schizosaccharomyces pombe. Nat Biotechnol. 2006;24: 841–7. doi: 10.1038/nbt1222 16823372 10.1038/nbt1222

[75] Nakaoka H, Wakamoto Y. (2017) Data from: Aging, mortality and the fast growth trade-off of *Schizosaccharomyces pombe*. Dryad Digital Repository. http://dx.doi.org/10.5061/dryad.s2t5t 10.1371/journal.pbio.2001109 PMC5478097 28632741

