# Supplementary material for: Aging, mortality, and the fast growth trade-off of Schizosaccharomyces pombe
Source: PLoS Biol. 2017 Jun 20;15(6):e2001109. doi: 10.1371/journal.pbio.2001109 (PMC5478097; doi:10.1371/journal.pbio.2001109)
Supplement: S1 Table — (DOCX) [file pbio.2001109.s001.docx]

| Table S1. Summary of measurements | | | | | | |
| --- | --- | --- | --- | --- | --- | --- |
| Medium | YE | | | | | |
| Temperature (˚C) | 28 | | 30 | | 34 | |
| Time window (min) | t ≥ 0 | t ≥ 3,000 | t ≥ 0 | t ≥ 3,000 | t ≥ 0 | t ≥ 3,000 |
| ROI | 5,974,355 | 3,958,645 | 4,942,955 | 3,242,927 | 4,590,656 | 2,795,040 |
| Time points | 3,395 | 2,395 | 3,569 | 2,569 | 3,361 | 2,361 |
| Time-lapse interval (min) | 3 | 3 | 3 | 3 | 3 | 3 |
| Observation period (min) | 10,185 | 7,185 | 10,707 | 7,707 | 10,083 | 7,083 |
| Total lineages | 1,844 | 1,656 | 1,571 | 1,334 | 1,618 | 1,265 |
| Extinct lineages | 715 | 527 | 831 | 594 | 1,000 | 647 |
| Surviving lineages | 1,129 | 1,129 | 740 | 740 | 618 | 618 |
| Division events | 93,567 | 62,368 | 89,058 | 56,717 | 90,360 | 54,072 |

| Table S1. Summary of measurements (continued) | | | | | | | | |
| --- | --- | --- | --- | --- | --- | --- | --- | --- |
| Medium | EMM | | | | | | | |
| Temperature (˚C) | 28 | | 30 | | 32 | | 34 | |
| Time window (min) | t ≥ 0 | t ≥ 7,500 | t ≥ 0 | t ≥ 3,000 | t ≥ 0 | t ≥ 7,500 | t ≥ 0 | t ≥ 9,000 |
| ROI | 6,977,258 | 2,257,803 | 6,954,885 | 5,054,392 | 6,310,760 | 1,817,395 | 77,07,359 | 1,883,024 |
| Time points | 3,785 | 1,285 | 3,949 | 2,949 | 3,703 | 1,203 | 4,111 | 1,111 |
| Time-lapse interval (min) | 3 | 3 | 3 | 3 | 3 | 3 | 3 | 3 |
| Observation period (min) | 11,355 | 3,855 | 11,847 | 8,847 | 11,109 | 3,609 | 12,333 | 3,333 |
| Total lineages | 1,770 | 1,573 | 1,654 | 1,534 | 1,785 | 1,346 | 1,979 | 1,344 |
| Extinct lineages | 304 | 107 | 388 | 268 | 573 | 134 | 791 | 156 |
| Surviving lineages | 1,466 | 1,466 | 1266 | 1266 | 1,212 | 1,212 | 1,188 | 1,188 |
| Division events | 74,420 | 23,953 | 86,849 | 62,613 | 86,746 | 23,255 | 86,355 | 21,378 |
