# Supplementary material for: Aging, mortality, and the fast growth trade-off of Schizosaccharomyces pombe
Source: PLoS Biol. 2017 Jun 20;15(6):e2001109. doi: 10.1371/journal.pbio.2001109 (PMC5478097; doi:10.1371/journal.pbio.2001109)
Supplement: S2 Table — Population doubling times estimated by growth curves of batch cultures are shown with standard errors. Mean generation time was calculated as the arithmetic mean. Uncertainty comes from the time resolution (3 min in these cases) of measurements. Population doubling time is generally shorter than mean generation time, and can be estimated using the Euler-Lotka equation (see Methods). (DOCX) [file pbio.2001109.s002.docx]

| Table S2. Comparison of cellular growth between batch culture and the microfluidic device | | | | | | | |
| --- | --- | --- | --- | --- | --- | --- | --- |
| Medium | YE | | | EMM | | | |
| Temperature (˚C) | 28 | 30 | 34 | 28 | 30 | 32 | 34 |
| Population doubling time (min)  (Batch culture) | 142±3 | 133±3 | 123±2 | 193±2 | 172±1 | 155±2 | 173±2 |
| ^a^Mean generation time (min)  (Microfluidic device) | 156±3 | 134±3 | 115±3 | 233±3 | 196±3 | 196±3 | 197±3 |
| ^b^Estimated Population doubling time (min)  (Microfluidic device) | 154 | 131 | 111 | 238 | 193 | 192 | 193 |
| Population doubling times estimated by growth curves of batch cultures are shown with standard errors. ^a^Mean generation time was calculated as the arithmetic mean. Uncertainty comes from the time resolution (3 min in these cases) of measurements. ^b^Population doubling time is generally shorter than mean generation time, and can be estimated using the Euler-Lotka equation (see Methods). | | | | | | | |
