# Supplementary material for: Aging, mortality, and the fast growth trade-off of Schizosaccharomyces pombe
Source: PLoS Biol. 2017 Jun 20;15(6):e2001109. doi: 10.1371/journal.pbio.2001109 (PMC5478097; doi:10.1371/journal.pbio.2001109)
Supplement: S3 Table — These data were used for the plots shown in Fig 3. (DOCX) [file pbio.2001109.s003.docx]

| Table S3. Summary of division and death rate estimations | | | | | | | |
| --- | --- | --- | --- | --- | --- | --- | --- |
| Medium | YE | | | EMM | | | |
| Temperature (˚C) | 28 | 30 | 34 | 28 | 30 | 32 | 34 |
| Division rate (x 10^-3^ min^-1^) | 6.52 | 7.61 | 8.94 | 4.29 | 5.26 | 5.35 | 5.28 |
| Division rate error (${\boldsymbol{2}\boldsymbol{S.E.}}_{\boldsymbol{division}}$) (x 10^-3^ min^-1^) | 0.006 | 0.006 | 0.010 | 0.012 | 0.006 | 0.022 | 0.016 |
| Death rate (× 10^-5^ min^-1^) | 6.30 | 8.35 | 11.04 | 1.96 | 2.65 | 3.93 | 3.75 |
| Death rate error ($\boldsymbol{2}\boldsymbol{\sigma}_{\boldsymbol{death}}$) (× 10^-5^ min^-1^) | 0.60 | 0.86 | 1.06 | 0.40 | 0.42 | 0.62 | 0.64 |
| Expected life span (generation) | 103 | 91 | 81 | 219 | 198 | 136 | 141 |
| Expected life span error ($\boldsymbol{\sigma}_{\boldsymbol{lifespan}}$) (generation) | 10 | 9 | 8 | 45 | 31 | 21 | 24 |
| Initial number of lineages | 1,759 | 1,396 | 1,339 | 1,581 | 1,595 | 1,380 | 1,352 |
| Observation period (min) | 7,182 | 7,704 | 6,819 | 3,852 | 8,844 | 3,606 | 3,330 |
| These data were used for the plots shown in Figure 3. | | | | | | | |
