# Supplementary material for: Aging, mortality, and the fast growth trade-off of Schizosaccharomyces pombe
Source: PLoS Biol. 2017 Jun 20;15(6):e2001109. doi: 10.1371/journal.pbio.2001109 (PMC5478097; doi:10.1371/journal.pbio.2001109)
Supplement: S4 Table — All the strains used in this study were constructed by crossing Bern collection K strains (L972 h- and L975 h+ derivatives). (DOCX) [file pbio.2001109.s004.docx]

| Table S4. List of fission yeast strains | | |
| --- | --- | --- |
| Strain | **Genotype** | **Source** |
| K6 | *h^-^ ade6-M375* | Bern Collection |
| K17 | *h^-^ ura4-D18* | Bern Collection |
| K128 | *h^+^ leu1-32 ade6-M26* | Bern Collection |
| HN0001 | *h^+^ leu1-32* | This study (K17 × K128) |
| HN0003 | *h^-^ leu1-32* | This study (K6 × HN0001) |
| HN0015 | *h^-^ leu1-32 ade6-M26* | This study (K128 × HN0003) |
| HN0025 | *h^-^ leu1-32*::*leu1^+^-Padh1-mVenus* | This study |
| HN0032 | *h^-^ leu1-32*::*leu1^+^-Phsp16-EGFP ade6-M26* | This study |
| HN0034 | *h^-^ leu1-32*::*leu1^+^-Phsp16-EGFP ade6-M26*::*ade6^+^-Padh1-mCherry* | This study |
| HN0041 | *h^-^ leu1-32*::*leu1^+^-Padh1-mCherry* | This study |
| HN0045 | *h^-^ leu1-32*::*leu1^+^-Padh1-mCherry hsp104-gfp* | This study |
| HN0053 | *h^-^ leu1-32 ade6-M26*::*ade6^+^-Padh1-mCherry* | This study |
| HN0060 | *h^-^ leu1-32*::*leu1^+^-Ptef-mNeonGreen-μNS ade6-M26*::*ade6^+^-Padh1-mCherry* | This study |
| HN0067 | *h^-^ leu1-32*::*leu1^+^-Ptef-mNeonGreen-μNS ade6-M26*::*ade6^+^-Padh1-mCherry hsp104*::*Kan^r^* | This study |
| HN0069 | *h^+^ hsp104*::*Kan^r^ leu1-32* | This study |
| HN0070 | *h^+^ hsp104*::*Kan^r^ leu1-32::leu1^+^-nmt1P41-mCherry-μNS* | This study |
| All the strains used in this study were constructed by crossing Bern collection K strains (*L972 h^-^* and *L975 h^+^* derivatives). | | |

*Padh1*: adh1 promoter that spans from −800 to −54 of the *adh1^+^* gene. The primers for cloning the promoter region were

5′-AATGGCATGCCCTACAACAACTAAGAAAAT-3′

and 5′-AGCTCATATGGAATTCTCTTGCTTAAAGAAAAGCGAAG-3′

Underlined bases indicate *Sph* I and *Nde* I sites used for cloning.

*Phsp16*: hsp16 promoter that spans from −1226 to −4 of the *hsp16^+^* gene. The primers for cloning the promoter region were

5′-ATCGGCATGCAAGAGCGATAGCTTCCGTCG-3′ and 5′-ATCGCATATGTTAAAATTTAAACAATTGCG-3′

Underlined bases indicate *Sph* I and *Nde* I sites used for cloning.

*Ptef*: TEF promoter upstream of Kanamycin resistance gene in pFA6a-KanMX6. The primers for cloning the promoter region were

5′-GTCGAGGAGAACTTCGACATGGAGGCCCAGAATACCCTCC-3′

and 5′-AGCTCATATGGGTTGTTTATGTTCGGATGTGATGTGAGA-3′

For tagging GFP at the C-terminus of Hsp104, 3’ end of the ORF region (2192 to 2716) and 3’ UTR region (2719 to 3220) were amplified using the following primers:

5′-TTATGACTTCTAACTTGGGCGCT-3′ (forward primer for the 3’ end of the ORF)

5′-TTAATTAACCCGGGGATCCGTTCCAATTCTTCATCATTAACATCGTCG-3′(reverse primer for the 3’ end of the ORF)

5′-GTTTAAACGAGCTCGAATTCATGTATTTCAAATGAGAAAGTTCTAACGA-3′ (forward primer for the 3’ UTR)

5′-GAGAACTGGAATGCGTATTAGTATGCAA-3′(reverse primer for the 3’ UTR)

Underlined bases indicate the pFA6a sequences.

For *hsp104^+^* deletion, 5’ UTR (-500 to -1) and 3’ UTR sequences (2719 to 3220) of the gene was amplified using the following primers:

5′-ATACACTACGTAATACGCAGATCATTTC-3′ (forward primer for the 5’ UTR)

5′-TTAATTAACCCGGGGATCCGATCGAGTTATATTATTATAGGAATTTTTA-3′ (reverse primer for the 5’ UTR)

5′-GTTTAAACGAGCTCGAATTCATGTATTTCAAATGAGAAAGTTCTAACGA-3′(forward primer for the 3’ UTR)

5′-GAGAACTGGAATGCGTATTAGTATGCAA-3′(reverse primer for the 3’ UTR)

Underlined bases indicate the pFA6a sequences.
