# Supplementary figures and images for: Aging, mortality, and the fast growth trade-off of Schizosaccharomyces pombe
Source: PLoS Biol. 2017 Jun 20;15(6):e2001109. doi: 10.1371/journal.pbio.2001109 (PMC5478097; doi:10.1371/journal.pbio.2001109)

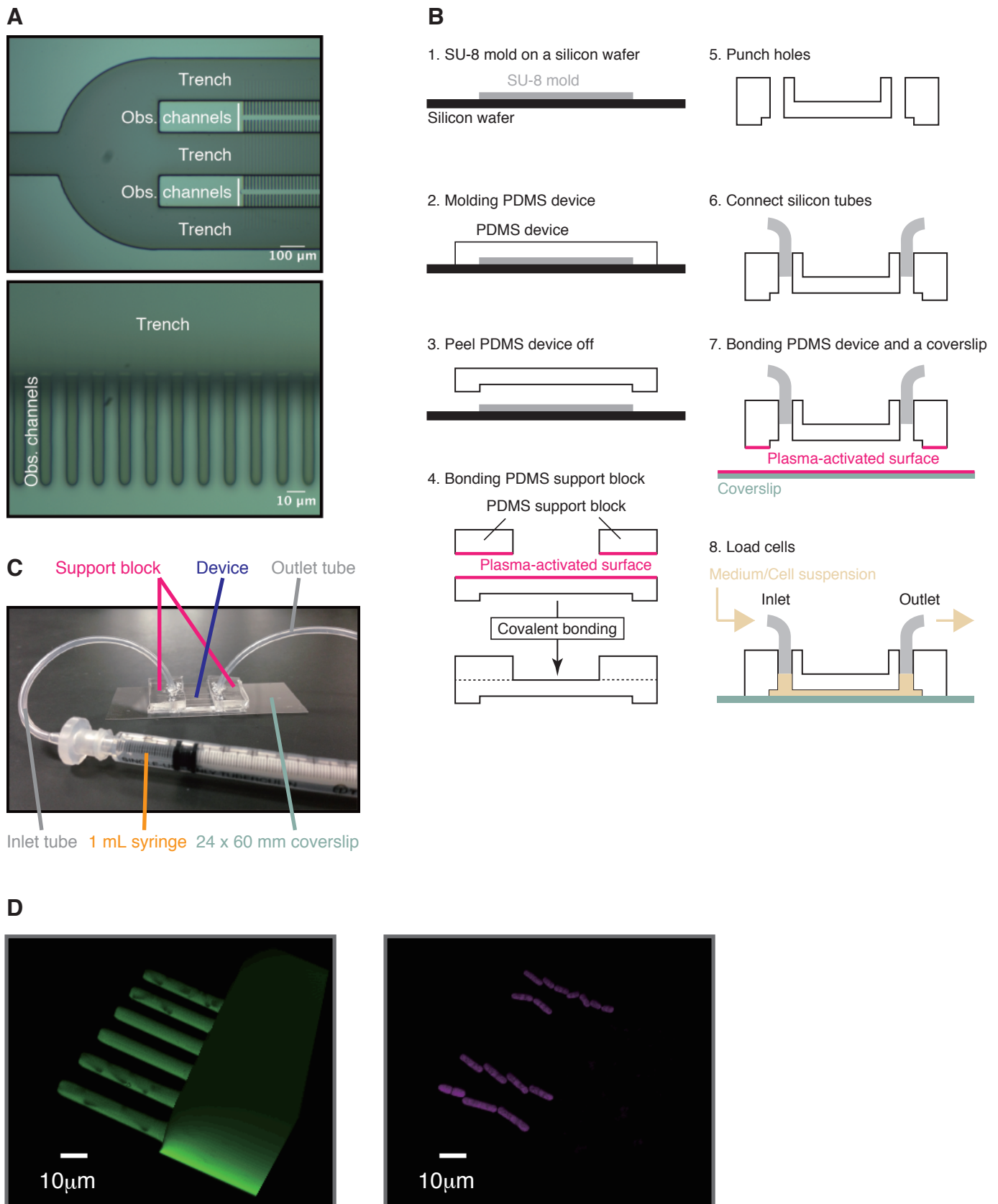

**Fig. S1**

Supplement: S1 Fig — (A) Microscopic images of a SU-8 mold on a silicon wafer. (Top) An image showing three trenches and four observation channel arrays. (Bottom) An enlarged view of one observation channel array. (B) Fabrication and assembly procedures for the PDMS microfluidic device. (C) A illustration of the microfluidic device connected to silicon tubing and a sample loading syringe. (D) A 3D view of the device and loaded cells reconstructed from confocal microscopic images. Cells expressing mCherry were delivered into the device with medium containing 3 μM fluorescein. Green fluorescence (left) and red fluorescence (right) images are presented. A 3D reconstruction was achieved using an ImageJ 3D viewer plug-in. (PDF) [file pbio.2001109.s005.pdf]

**A**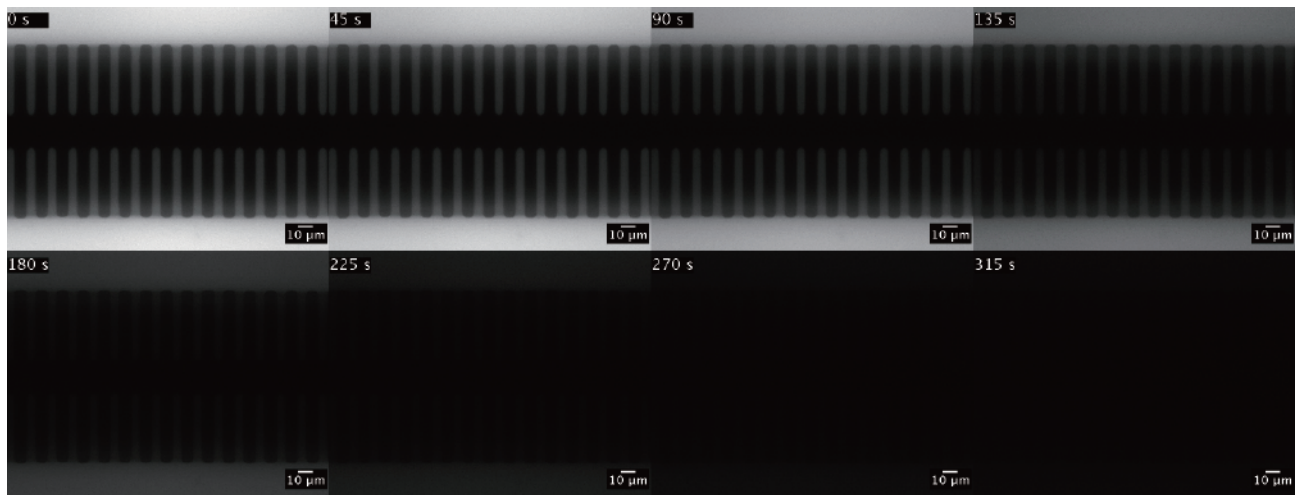**B**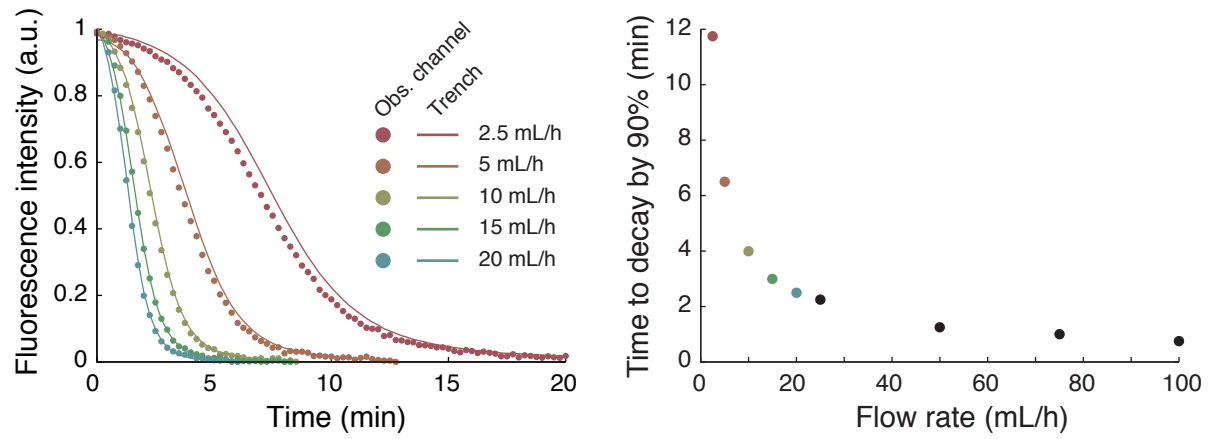**C**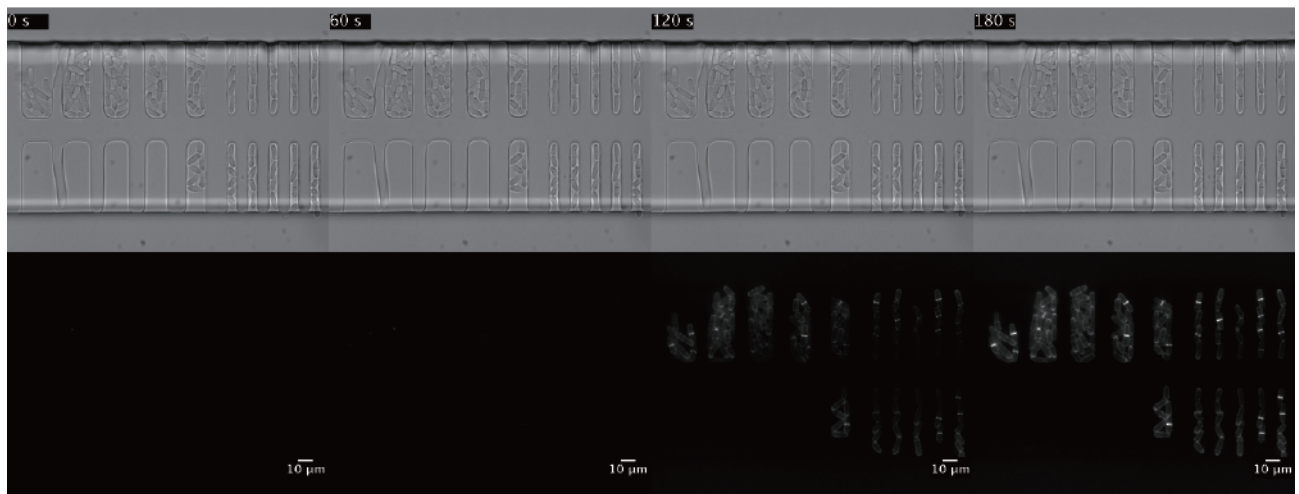**Fig. S2**

Supplement: S2 Fig — (A) Time-lapse fluorescence images showing fluorescein removal. The device was first filled with YE containing 3 μM fluorescein, and then YE medium without fluorescein was supplied at a flow rate of 10 mL/h. (B) Fluorescein removal at different flow rates. The same experiments as in (A) were performed at various flow rates, and the decay of fluorescence intensity was plotted against time (left). Fluorescence intensity was normalized to the values at t = 0. The points indicate the decay of fluorescence in the observation channels, and the lines indicate this decay in trenches. The 90% decay time was less than 5 min when the flow rate was greater than 10 mL/h (right). The experiments described in the main text were performed at 10–15 mL/h. (C) Quick introduction of fluorescent dye into observation channels. After loading of cells, YE medium containing 20 μg/mL of Calcofluor White Stain (Sigma-Aldrich), which stains cell walls, especially septa, was supplied at a flow rate of 10 mL/h. Cells in both narrow and wide observation channels were stained with the same kinetics, suggesting that the medium was effectively supplied even in the presence of cells in the thin observation channels. It is also of note that the cells at the ends of the channels were stained as efficiently as those at the exits of the channels. (PDF) [file pbio.2001109.s006.pdf]

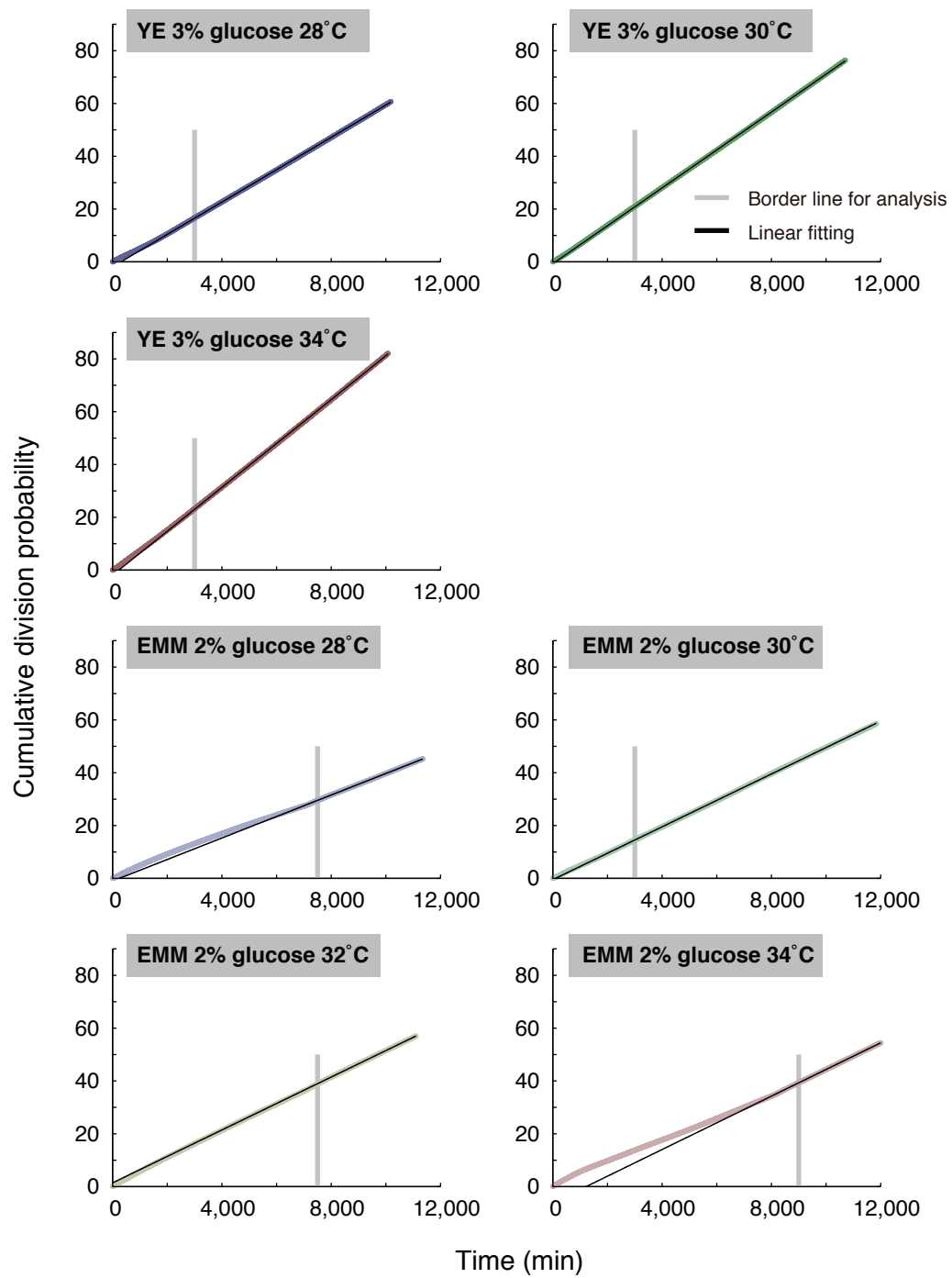

Fig. S3

Supplement: S3 Fig — Linear fitting was performed using the time window after the gray vertical lines, where stable cellular growth was achieved. (PDF) [file pbio.2001109.s007.pdf]

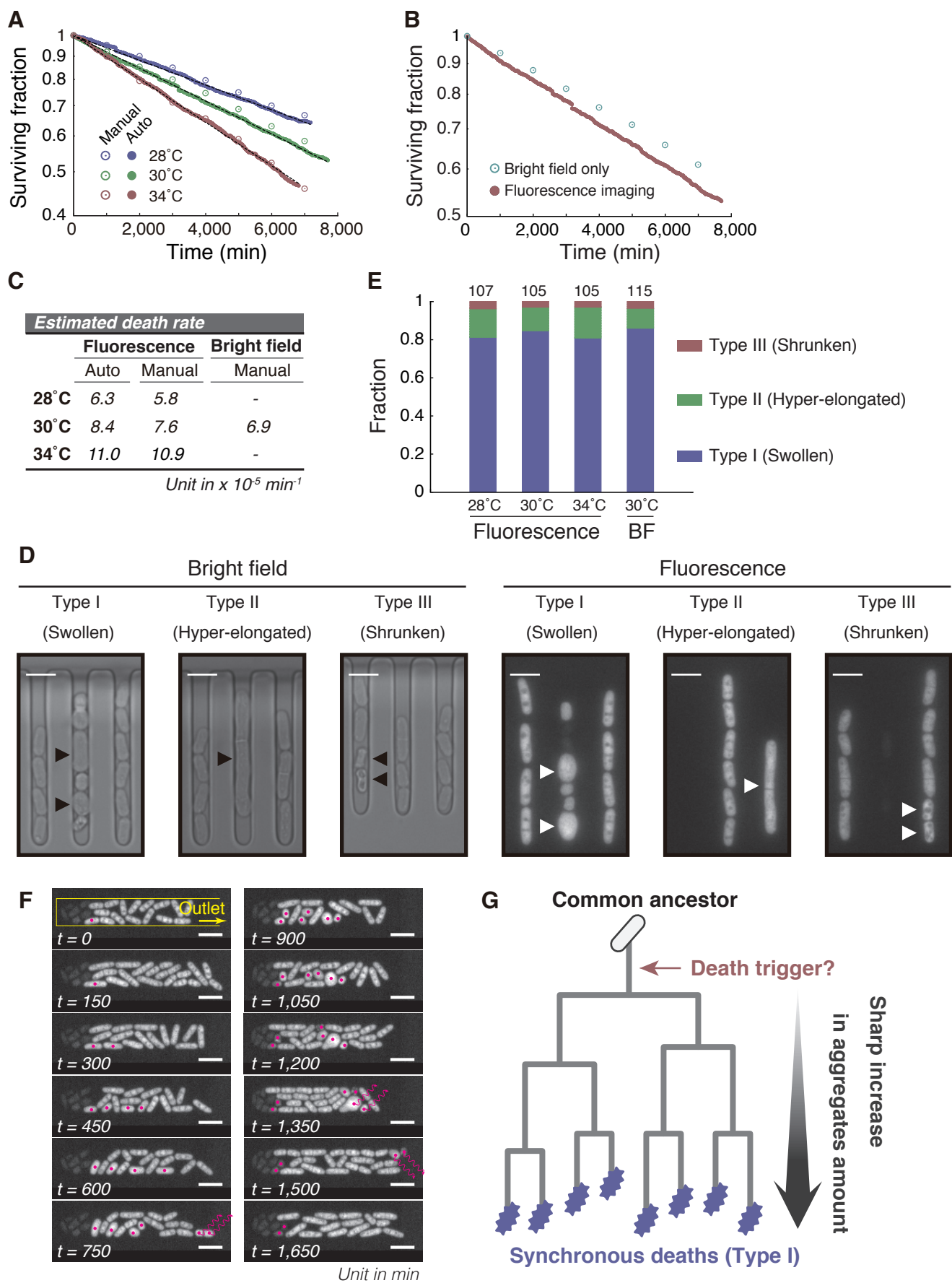

Fig. S4

Supplement: S4 Fig — (A) Comparison between automated and manual estimations of death rates. To validate the estimations of death rates based on our automated cell death detection algorithm, the survival curves were compared with those obtained by manual inspections. For manual estimations of death rates, surviving old-pole cells were counted by eye every 1,000 min at all positions, and the exponential decay rates were estimated by least squares fitting. Although the automated algorithm might overestimate death rates, the differences between the automated and manual estimations were minor (at most 10%), and the survival curves were similar. (B) Photodamage effects on cell deaths. To evaluate photodamage induced by the excitation light used in fluorescence imaging, a long-term time-lapse experiment was performed with only bright-field imaging in YE at 30°C. The surviving fraction was plotted against time (light blue open circles) and compared with that in fluorescence imaging (red closed circles) in the same medium and temperature. The estimated death rate in fluorescence imaging was 20% higher (8.35/6.94 = 1.20) than that in bright-field imaging. (C) Summary of the estimated death rates in (A) and (B). (D) Classification of the death modes. Cells exhibited three types of morphological changes before death: swollen, hyper-elongated, and shrunken. Examples of bright-field or fluorescence microscopic images of these three types are shown. Dead/dying cells are indicated by arrowheads. Scale bars indicate 10 μm. (E) Fraction of each death mode in different environments. Approximately 80% of spontaneous cell deaths are classified as Type I (swollen and synchronous) in all the tested environments. (F) Synchronous cell deaths in other microfluidic device. The device has the same architecture as the Mother Machine-type device described in the main text, except that the observation channels are wider and can accommodate more cells. Progenies of a common ancestor cell (indicated by a magenta circl [file pbio.2001109.s008.pdf]

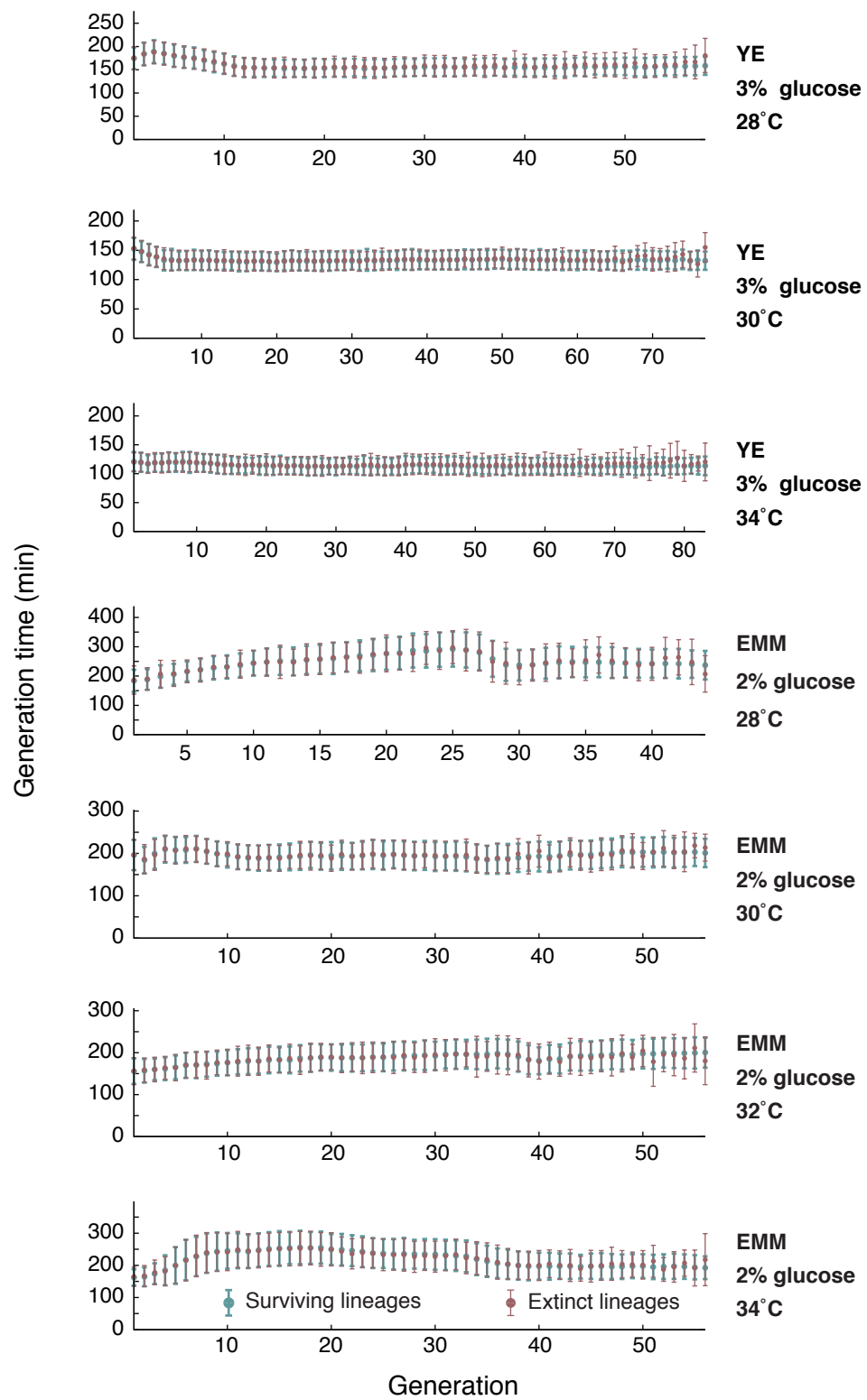

**Fig. S5**

Supplement: S5 Fig — Mean generation times for each generation are plotted with error bars representing standard deviations. (PDF) [file pbio.2001109.s009.pdf]

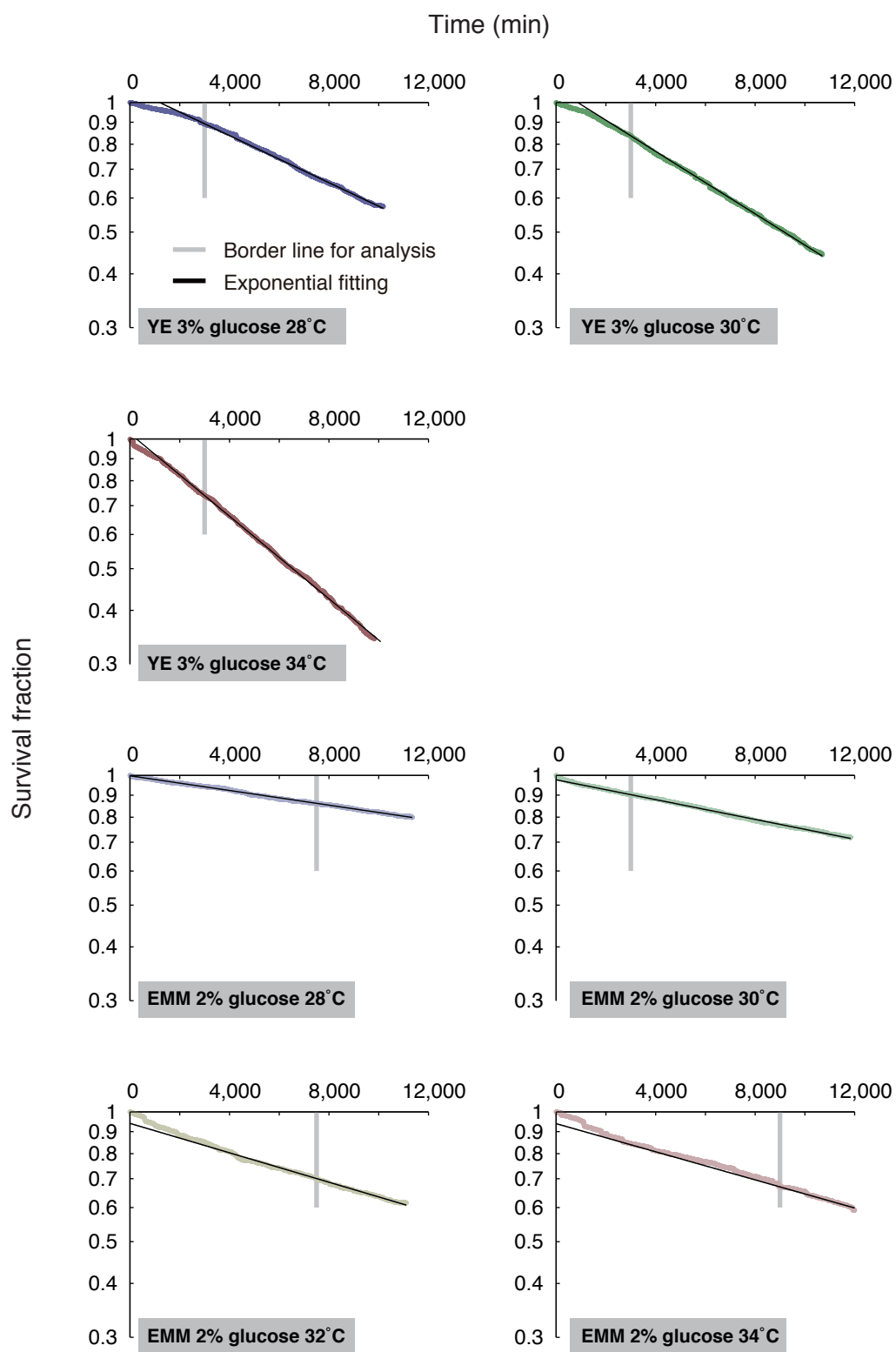

Fig. S6

Supplement: S6 Fig — The surviving fractions of the old-pole cell lineages were plotted against time in a semi-log plot. Death rates were stable in the time windows after the gray vertical lines, where stable growth was achieved (see S3 Fig). (PDF) [file pbio.2001109.s010.pdf]

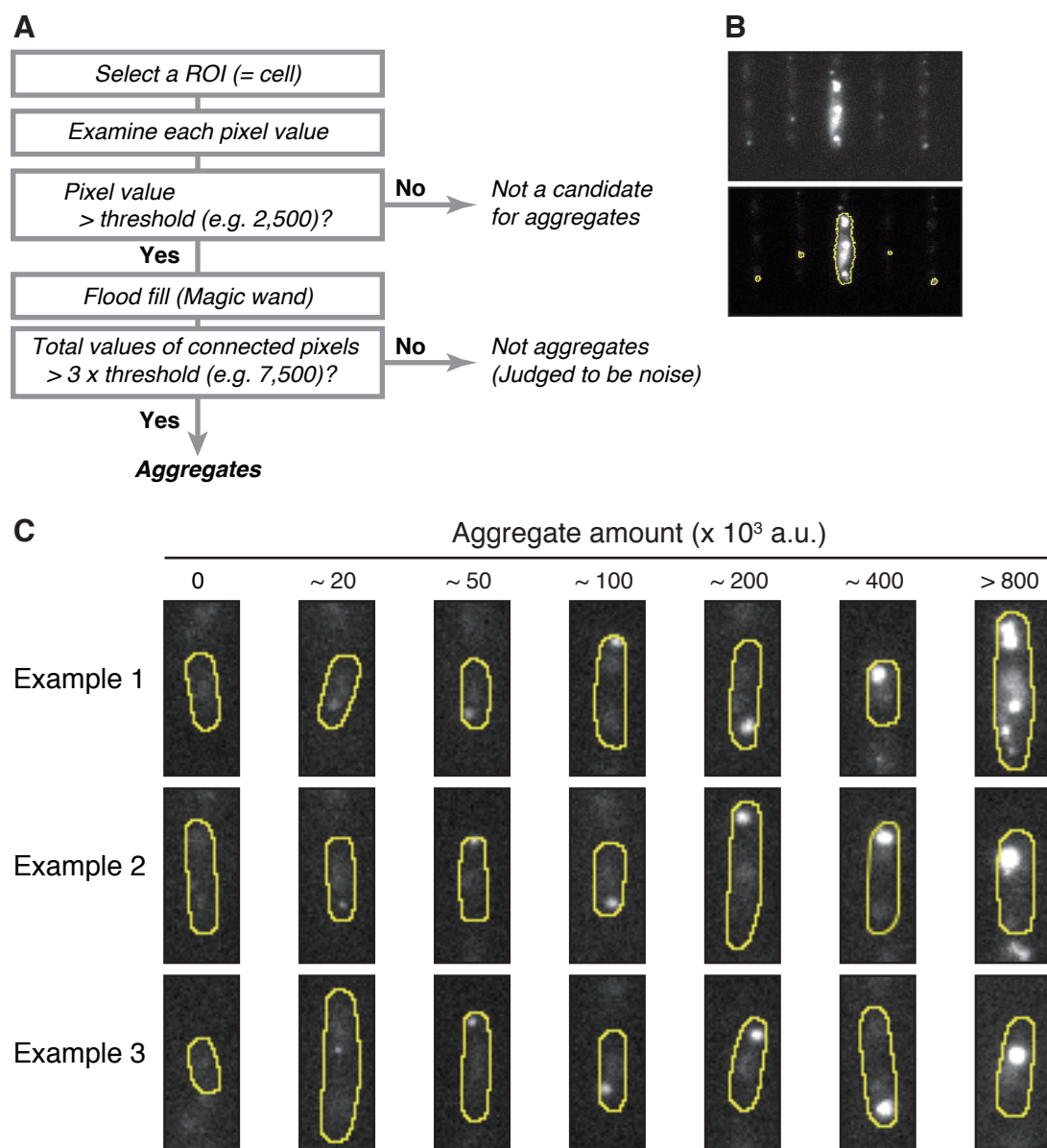

**Fig. S7**

Supplement: S7 Fig — (A) An algorithm of identification of protein aggregate foci. (B) An example image of identified aggregate foci. The top image shows the representative Hsp104-GFP fluorescence, and the bottom image illustrates the identified aggregates outlined in yellow. (C) Representative images of cells with different amounts of aggregate. (PDF) [file pbio.2001109.s011.pdf]

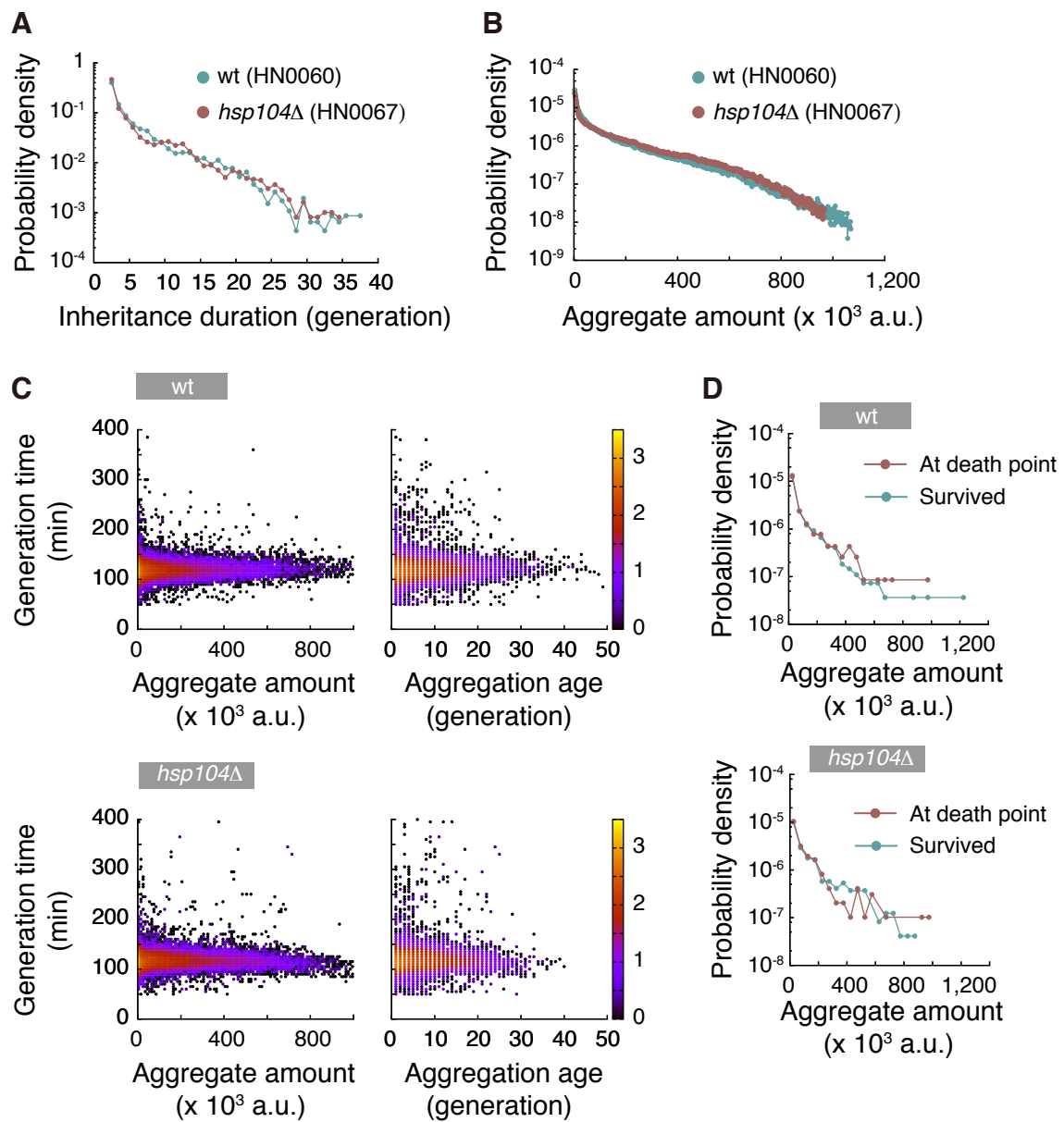

Fig. S8

Supplement: S8 Fig — (A) Distributions of inheritance duration of mNeonGreen-μNS aggregate. (B) Distributions of aggregate amount of mNeonGreen-μNS. (C) Density plots showing the relations between generation time and aggregate amount (left) and between generation time and aggregation age (right). The plots for both wildtype and hsp104Δ strain are presented. (D) Distributions of mNeonGreen-μNS aggregate amounts at death points (red) and at the end of the measurements for the surviving lineages (blue). The left plot shows the result for wildtype; and the right plot for hsp104Δ strain. (PDF) [file pbio.2001109.s012.pdf]
